# Peer support of complex health behaviors in prevention and disease management with special reference to diabetes: systematic reviews

**DOI:** 10.1186/s40842-017-0042-3

**Published:** 2017-05-25

**Authors:** Edwin B. Fisher, Renée I. Boothroyd, Emily A. Elstad, Laura Hays, Amy Henes, Gary R. Maslow, Clayton Velicer

**Affiliations:** 1 Peers for Progress, Gillings School of Global Public Health, University of North Carolina, Box 7440, Chapel Hill, NC 27599-7440 USA; 2 Department of Health Behavior, Gillings School of Global Public Health, University of North Carolina, Box 7440, Chapel Hill, NC 27599-7440 USA; 3 Frank Porter Graham Child Development Institute, University of North Carolina, Chapel Hill, NC USA; 4 American Institutes for Research, Chapel Hill, NC USA; 5 Indiana University School of Nursing, Indianapolis, IN USA; 6 RTI International, Research Triangle Park, NC USA; 7 Department of Pediatrics, Duke University, Durham, NC USA; 8 Department of Psychiatry and Behavioral Sciences, Duke University, Durham, NC USA; 9 National Public Relations and Communications, Kaiser Permanente, Oakland, CA USA

**Keywords:** Peer support, Prevention, Disease management, Social support, Community health workers

## Abstract

**Objectives:**

Examine Peer Support (PS) for complex, sustained health behaviors in prevention or disease management with emphasis on diabetes prevention and management.

**Data sources and eligibility:**

PS was defined as emotional, motivational and practical assistance provided by nonprofessionals for complex health behaviors. Initial review examined 65 studies drawn from 1442 abstracts identified through PubMed, published 1/1/2000–7/15/2011. From this search, 24 reviews were also identified. Extension of the search in diabetes identified 30 studies published 1/1/2000–12/31/2015.

**Results:**

In initial review, 54 of all 65 studies (83.1%) reported significant impacts of PS, 40 (61.5%) reporting between-group differences and another 14 (21.5%) reporting significant within-group changes. Across 19 of 24 reviews providing quantifiable findings, a median of 64.5% of studies reviewed reported significant effects of PS. In extended review of diabetes, 26 of all 30 studies (86.7%) reported significant impacts of PS, 17 (56.7%) reporting between-group differences and another nine (30.0%) reporting significant within-group changes. Among 19 of these 30 reporting HbA1c data, average reduction was 0.76 points. Studies that did not find effects of PS included other sources of support, implementation or methodological problems, lack of acceptance of interventions, poor fit to recipient needs, and possible harm of unmoderated PS.

**Conclusions:**

Across diverse settings, including under-resourced countries and health care systems, PS is effective in improving complex health behaviors in disease prevention and management including in diabetes.

**Electronic supplementary material:**

The online version of this article (doi:10.1186/s40842-017-0042-3) contains supplementary material, which is available to authorized users.

## Background

### Peer support of complex health behaviors in prevention and disease management: a review with emphasis on diabetes

 Peer support (PS) provided by “community health workers,” “lay health advisors,” “*promotores*,” “patient navigators,” “peer supporters,” and individuals with a number of other titles has been shown to play influential roles in health and the health care delivery system [1–4]. In particular, PS strategies can encourage appropriate regular care, can provide practical and emotional support for complex behaviors that are critical to staying healthy, and can help individuals cope with the stressors chronic diseases and conditions so often entail [5–15]. Calls-to-action and formal policy recommendations for the implementation of PS approaches [16–19] include numerous provision for community health workers in the US Affordable Care Act and a strong emphasis on Community Health Workers in the World Health Organization’s Global Health Workforce Alliance [20]. Programmatic and methodological challenges nevertheless limit what we know about PS approaches’ impact on health. Among these challenges, PS often takes on many definitions, roles, and forms [21–24] making it difficult to summarize or consolidate evidence across studies.

As detailed in the Methods and Results, a number of reviews have examined PS programs. However, most of these have focused on a specific health problem or challenge (e.g., promoting breastfeeding), or modality (e.g., telephone support). As detailed in this paper, we identified 24 reviews of PS interventions, of which 21 were focused on PS in a specific problem area of prevention or care, or a specific modality. The three that examined PS more broadly included that by Swider [1] published in 2002. That by Viswanathan and colleagues [2] limited its focus to PS through community health worker interventions to “create a bridge between community members, especially hard-to-reach populations, and the health care system” (p. 793). It found “moderate” evidence in impacts on knowledge, health behaviors, utilization, and cost/cost effectiveness. The third, by Gibbons and Tyus [3] limited its focus to US-based programs for those traditionally lacking access to care. It reported “efficacy in enhancing outcomes” across mammography, cervical cancer screening, and a variety of other health/prevention objectives. A fourth, more recent review by Perry and his colleagues in the 2014 *Annual Review of Public Health* [25] identified contributions of community health workers to basic health needs in low-income countries (e.g., reducing childhood undernutrition), to primary care and health promotion in middle income countries, and to disease management in the United States and other countries with developed economies.

In this present review, we sought to identify reports of PS interventions from around the world, addressing a wide variety of prevention and health objectives entailing sustained and complex behavior change (e.g., promoting diabetes management, but not flu shots), and using a broad definition of PS entailing assistance and encouragement for those behaviors as well as linkage to appropriate care. We then extended that review in two ways. We included a review of reviews of peer support across the same broad range of complex behavior change, and we updated the review through the end of 2015 focusing on diabetes prevention and management, diabetes being both a major source of global disease burden and a model for chronic disease prevention and care in general.

#### The rationale for peer support

Diabetes provides also important models for addressing the major challenge to all of health and health care: sustaining preventive and disease management behaviors. This is reflected, for example, in standards for diabetes education and support of the American Diabetes Association and American Association of Diabetes Educators. They call not only for diabetes self management education, but also call for and distinguish diabetes self-management ***support*** to help those with diabetes “implement and sustain the behaviors needed to manage their illness.” [26, 27] In spite of recognition that “chronic disease needs chronic care,” the research literature does not reflect the importance of ongoing support for self management. For example, a search of PubMed (14 November, 2016) for articles with cognates of **“**diabetes**”** and **“**self-management**”** in their titles or abstracts yielded 3783 responses. Narrowing the search by adding cognates of “sustain” or “maintain” or “ongoing” yielded 533, 14.1%.

There are a number of reasons PS may be helpful to ongoing support for self management. These include PS providing time, rehearsal and problem solving around key health behaviors, and emotional and social support and encouragement. To focus on this, we have limited the first part of this review to ***support*** for and ***maintenance*** of ***complex*** health ***behavior change***, such as in chronic disease management or extensive prevention efforts as in weight loss or smoking cessation, problems for which the need for ongoing support to ***sustain behavior change*** is central. Of course, the extended review of peer support in diabetes shares these emphases because of the nature of diabetes prevention and management.

We have not included PS interventions addressing relatively isolated or single behaviors, e.g., screening or inoculations or short-term medication compliance. Although PS may be very valuable in these areas, [28] behaviorally these represent very different tasks. Additionally, community organization or capacity building were not inclusion criteria. Again, it is not that these are not often important roles, especially for many community health workers and *promotores de salud,* [11] but that we sought to focus the review on the support of individuals or groups of peers for sustained, complex health behaviors.

The review was conducted as a project of Peers for Progress (peersforprogress.org), a program in the Gillings School of Global Public Health at the University of North Carolina-Chapel Hill that focuses on peer support in health, health care and prevention [29–32]. Peers for Progress has pursued a strategy of defining peer support not by specific implementation protocols or details but according to four “key functions of support.” [33, 34] This follows a strategy of “standardization by function, not content.” [35, 36] The four key functions are: (i) assistance in daily management; (ii) social and emotional support to encourage management behaviors and coping with negative emotions; (iii) linkage to clinical care and community resources; and (iv) ongoing availability of support because chronic disease is for the rest of one’s life [34]. With tailoring according to needs and strengths of a specific setting or health challenge, these become a template for planning and evaluating peer support programs [30, 33]. As described further below, they were also used to identify papers for inclusion in this review.

The purpose of this review, then, is to characterize PS programs from around the world and their ability to promote sustained, complex health behaviors and, especially, such behaviors in diabetes prevention and management. Due to the variety of specific intervention approaches, designs, and outcomes of the literature reviewed, meta-analytic approaches were not employed. Instead, the systematic review characterizes studies identified and summarizes their findings.

## Methods

As appropriate, the Methods, Results and Discussion are organized according to the PRISMA checklist for reporting results of systematic reviews [37]. PS was defined as social support shared among or provided by nonprofessionals and that includes emotional, social and practical assistance necessary for sustaining complex health behaviors such as chronic disease management, management of chronic psychological problems such as depression, or risk reduction such as in smoking cessation or obesity management. PS was not limited to face-to-face contact but could include phone calls, text messaging, group meetings, home visits, or shared activities such as physical activity or grocery shopping. PS could be provided by nonprofessionals with a number of titles, e.g. community health worker, *promotora*, *doula*, navigator, etc.

## Information sources and search in initial review

We conducted a systematic literature search of PubMed for articles in English using cognates of any or all of the following in their titles or abstracts: “Coach,” “Community Health Aide,” “Community Health Promoter,” “Community Health Representative,” “Community Health Worker,” “*Consejeras*,” “*Doula*,” “*Dumas*, “*Embajadores*,” “Health Advocate,” “Health Worker,” “Lay Health Adviser,” “Lay Health Worker,” “Natural Helper,” “Outreach Worker,” “Peer Educator,” “Peer Provider,” “Peer Support,” or “*Promotora*.” To reflect the current state of the art in the field, papers published before January 1, 2000 were not included. The time period for the review extended through July 15, 2011. Further search limits were set to filter by trial type, including randomized and/or controlled clinical trials, reviews, and evaluation studies. In addition to search of PubMed, articles were also obtained from the citations of papers included in the final sample.

## Eligibility - study selection

Initially, paper titles and abstracts were reviewed by one author (EE or LH) and were excluded outright for: discussion (not research) papers, papers unrelated to our topic (e.g., “coach” in the context of sports or professional development), or papers without an abstract. Studies identified as potentially relevant were retrieved in full text as necessary and screened for inclusion by one of the authors (EE or LH) and reviewed by a group of at least three of the authors who reached consensus as to whether to include the paper in the review.

Drawing from the four key functions of PS as used by Peers for Progress, described above, inclusion criteria were:

- Ongoing support from a nonprofessional
- Assistance or consultation in applying management or behavior change plans in daily life and/or
- Social and emotional support directed toward emotional status, well being, or quality of life, and/or
- Encouragement of recommended care.

Studies were excluded if the intervention was conducted by a professional. This was operationalized as post-baccalaureate training in a health profession. For example, one paper was excluded because support for physical activity was provided by graduate students in kinesiology.

To achieve the focus on PS for complex health behaviors extended over time, studies were also excluded if the PS program:

1. Addressed temporally isolated behaviors or single behaviors (e.g., mammography, vaccination) rather than complex behaviors extended over time
2. Was limited to a fixed number of group programs or classes or peers implementing a highly scripted information program. Group programs taught or facilitated by nonprofessionals are important strategies of health promotion but do not constitute ongoing PS for sustaining health behaviors of the sort the present review was intended to evaluate.
3. Involved non professionals in roles limited to assisting others, such as in setting up rooms, distributing materials in classes, etc.
4. Indicated PS as the outcome variable rather than the independent variable, it being the intent of this review to assess the *effects of* PS.

Additional exclusion criteria were:

5. No statistical tests of significance of changes observed, rendering indistinguishable reports of changes versus nonsignificant changes.
6. Control or comparison conditions that provided a substantial amount of social or PS that may have masked or obscured the effects of social/peer support. This included, e.g., studies of group interventions in which all conditions included encouragement of social support among group members. However, given that PS is intrinsic to almost any group intervention and thus to avoid shrinking the pool of papers substantially, we retained several papers, (e.g., [38]) that included support in all conditions but which focused evaluation on additional PS in an experimental condition.

Studies were not excluded based on quality, except insofar as presentation prevented assessing inclusion and exclusion criteria and/or reports of outcomes.

## Data collection and abstraction

Informed by the Center for Disease Control’s Community Guide to Preventive Services, [39] a data abstraction matrix was devised to capture relevant information regarding the methods used and outcomes achieved by each study. Using the data abstraction tool, each article was reviewed by one of the authors and findings discussed among a group of at least three of the authors, consulting the original text as needed. Final coding was by consensus.

## Extended search for studies related to diabetes management

Due to the global burden of diabetes and its status as a model for prevention and management of other chronic diseases, the survey of papers related to diabetes was extended through December 31, 2015. This used the same syntax as for the initial, broader review with the addition of the specification of “diabetes” as a keyword in the search. The methods and criteria for identifying and reviewing papers were the same as those for the broader review with the exception that they were carried out by one of us (EF).

## Data items

### Categorization of designs

Studies were categorized according to their design as: (a) Randomized Controlled Trials (RCTs) (including cluster randomized designs), (b) Other Controlled Designs (including quasi-experimental and multiple baseline designs), (c) Within Groups, Pre-Post designs, and (d) Other designs such as analyses of uncontrolled program records, non-equivalent comparison groups, or comparisons to those offered a program but who chose not to join. Allocation was conservative. For example, a comparison to “those who requested service and were not contacted” [40] was characterized as quasi-experimental in its published paper but was categorized in this review as “Other.”

### Categorization of measures employed

Studies were coded according to their outcome measures as follows:

- Objective Measures included measures such as Hemoglobin A1c (HbA1c) measure of blood glucose, body-mass index (BMI), or blood pressure as assessed by research or clinical staff but not by self report. This also included data from computer-based medical or hospital records such as hospitalization data or data registries.
- Standardized Measures included validated scales (e.g. Beck Depression Inventory) or survey strategies (e.g., Structured Clinical Interview for psychological/psychiatric disorders) and included standardized measures of behavioral outcomes such as dietary patterns, medication adherence, or self efficacy (e.g., Morisky Medication Adherence Scale, Diabetes Management Self-Efficacy Scale).
- Non-Standardized Measures included self-report measures or audits for which no validation studies or procedures were cited or described. This category also included manual or chart audits of clinical records from which, in contrast to computerized data bases or registries, extraction of data is subject to interpretation and other human errors.

A number of studies provided challenges to this coding of measures. In cases in which the proper coding of a study’s outcome measure was unclear, the study was coded in the ‘weakest’ type, i.e., Non-Standardized weaker than Standardized weaker than Objective. For example, a study of impacts on breastfeeding utilized computer-based administrative data from the Women’s Infants and Children’s program in Michigan [40]. On the one hand, this might be coded as Objective because the data were drawn from an administrative database. However, because the *source* of the data in that administrative database appeared to be case workers’ notes, the study was coded as Non-Standardized.

### Categorization of outcomes

Outcomes were categorized as: Significant Between Group differences (SBG) or Significant Within Group or pre-post differences (SWG) indicating effects of PS, Nonsignificant (NS), or Counter indicating negative impacts of PS. Based on these categories, studies were then categorized according to their strongest findings. That is, a paper with one Significant Between Group difference and one Significant Within Group difference would be placed in the former category. Because of the variety of designs and multidimensional nature of many evaluations, it was not possible to categorize studies according to their primary or hypothesized outcomes.

### Review of reviews

In addition to the original research papers identified through the search described above, reviews of PS interventions were captured using parallel search strategies and from the references of the research studies identified. Many of these reviews disaggregated studies into specific categories such as by type of outcome, e.g., knowledge, health behaviors, clinical measures, quality of life. For each of these categories of each review, the percentages of studies showing a significant effect of PS was documented.

## Results

### Study selection

**Fig. 1:**
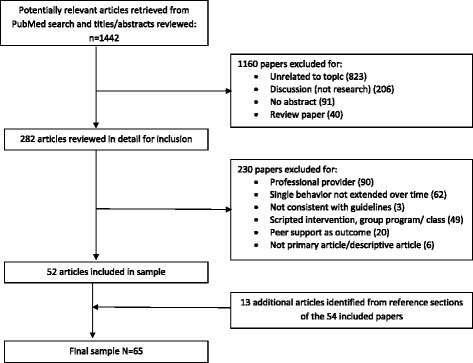
Identification, Exclusion and Selection of Studies for Review: Jan 1, 2000 – July 15, 2011

The literature search for the initial review identified 1442 peer reviewed articles. The process of selection of relevant articles is displayed in Fig. 1. In the first iteration of article selection, 1160 articles were excluded because their titles and/or abstracts indicated that they were not research/intervention papers. Of the remaining 282 articles, 230 were excluded based on the specifics of these programs (as described in the Methods and displayed in Fig. 1). This selection process yielded 52 articles describing the effect of PS programs on complex behavioral health outcomes. An additional 13 articles were identified from the reference sections of these 52 papers, yielding a final sample of 65.

### Study characteristics

Details of all 65 studies are available in Additional file 1, “Details of Studies from Systematic Review of Peer Support.”

The 65 studies in the final sample represented eight countries: 34 studies from the United States, seven from Canada, four from each of Bangladesh, England, Pakistan, and Scotland, and one from each of Australia, Brazil, Denmark, Ireland, Mozambique, New Zealand, South Africa, and Uganda. Fifty three were from World Bank designated high-income countries and 12 from low-income, low-middle, and high-middle-income countries.

As detailed in Table 1, PS interventions addressed a variety of health conditions and both prevention and management.

**Table 1:** Type of health problem addressed and categorization of focus on prevention or disease management for 65 studies included in review

Type of Health Problem Addressed	Prevention	Management
Addiction: Drug, Alcohol, Cigarette Smoking	0	3
Cardiovascular disease, including Heart Failure and general prevention through any or all of diet, exercise, PA, BP mgmgt	5	5
Diabetes, including support for parents of children with diabetes	2	7
HIV/AIDS	2	4
Maternal & Child Health: Pregnancy, Childbirth, Pre- and Post-natal care, including breastfeeding in “Prevention or Health Promotion”	15	2
Mental Health, including post-partum depression (3)	2	6
Other Chronic Disease	0	12
• Asthma, including support for parents of children with asthma	0	6
• Cancer, including breast cancer (2), survivorship	0	4
• Chronic Fatigue Syndrome	0	1
• COPD	0	1
Totals:	26	39

As detailed in Table 2, 48 of the studies used RCTs (including cluster randomized designs), four used other controlled designs (such as multiple baseline), seven used within-groups, pre-post designs, and seven used a variety of other designs without systematic control procedures (analyses of uncontrolled program records, [41] non-equivalent comparison groups, [40, 42] comparisons to those offered a program but who chose not to join, [43] comparisons to sites in another city or region, [44, 45] and a pilot of a study under development [46]).

**Table 2:** Outcomes of peer support interventions disaggregated by type of design and type of measure. (Percentages within each category of design and measure in parentheses)

Outcome	Randomized controlled trials			Other controlled designs			Within groups, pre-post designs			Other designs			Totals
	Objective	Standardized	Nonstandardized	Objective	Standardized	Nonstandardized	Objective	Standardized	Nonstandardized	Objective	Standardized	Nonstandardized	
Significant between groups	6 (60.0)	23 (74.2)	5 (71.4)	1 (100.0)	1 (100.0)	1 (50.0)	−	−	−	0	1 (50.0)	2 (50.0)	40 (61.5)
Significant within groups	2 (20.0)	3 (9.7)	0	0	0	1 (50.0)	0	3 (75.0)	2 (100.0)	1 (100.0)	1 (50.0)	1 (25.0)	14 (21.5)
Nonsignificant	2 (20.0)	3 (9.7)	2 (28.6)	0	0	0	0	1 (25.0)	0	0	0	1 (25.0)	9 (13.8)
Counter	0	2 (6.5)	0	0	0	0	0	0	0	0	0	0	2 (3.1)
Totals	10	31	7	1	1	2	0	4	2	1	2	4	
		48			4			6			7		65

### Synthesis of results

#### Effects of PS

Table 2 presents the results of all studies disaggregated by type of design, type of outcome measure, and outcome. Across all 65 studies included in this review, 40 (61.5%) reported significant, between-group differences favoring PS. An additional 14 (21.5%) reported significant within-group changes indicating effects of PS among those who received it. Together then, 54 of all 65 studies (83.1%) reported significant effects of PS. Nine studies (13.9%) reported no significant effects of PS, and two studies (3.1%) reported results counter to effects of PS.

Aggregating all 48 RCTs in Table 2, a total of 34 (70.8%) reported significant between-group differences, another 5 (10.4%) reported significant within group differences, so that a total of 39 of the 48 (81.2%) reported significant between- or within-group differences favoring PS. Disaggregating by type of measure, 6 of 10 RCTs that used objective measures reported significant between-group differences and another two of these ten reported significant within-group differences. Thus, a total of 8 of 10 (80%) RCTs using objective measures reported significant between- or within-group differences indicating effects of PS. Adding in those using standardized measures, a total of 34 of 41 (82.9%) RCTs using objective or standardized measures reported significant between-group (29 of 41, 70.7%) or within-group (5 of 41, 12.2%) differences indicating effect of PS.

Table 3 presents the data aggregating across RCT and Other Controlled Designs. Aggregating also across both Objective and Standardized measures, 31 of 43 (72.1%) of studies reported significant between-condition effects favoring PS, and an additional 5 (11.6%) reported significant within-condition effects. Combining these, 36 of 43 or 83.7% of RCT or Other Controlled Designs using objective or standardized measures reported significant effects of PS.

**Table 3:** Outcomes of randomized controlled trial and other controlled design evaluations of peer support interventions by type of measure. (Percentages within each category of design and measure)

	RCT and other controlled designs			Totals
Outcome	Objective	Standardized	Nonstandardized	
Significant between groups	7 (63.6)	24 (75)	6 (66.7)	37 (71.2)
Significant within groups	2 (18.2)	3 (9.4)	1 (11.1)	6 (11.5)
Nonsignificant	2 (18.2)	3 (9.4)	2 (22.2)	7 (13.5)
Counter	0	2 (6.3)	0	2 (3.8)
Totals	11	32	9	52

Examination of Table 2 indicates little variation among patterns of findings across type of designs. Among RCTs, 81.3% (39 of 48) found significant between- or within-group differences indicating effects of PS, as did 100% (4 of 4) of Other Controlled Designs, 83.3% (5 or 6) of Within-Group designs, and 85.7% (6 of 7) of Other designs. Disaggregating by type of design and type of measure, the lowest percentage of studies reporting evidence of effects of PS was 71.4% among the seven reporting RCTs with nonstandardized outcome measures. It should be noted that seven of the nine studies reporting nonsignificant findings and both of the studies reporting findings counter to the effects of PS were RCTs.

#### Type of health problem

Eighteen of the 26 studies addressing prevention (69.2%) reported significant between-group differences and 6 (23.1%) reported significant within-group changes reflecting effects of PS (total = 92.3%). Among the 39 addressing management, 22 (56.4%) reported significant between-group differences and 9 (23.1%) reported significant within-group changes favoring PS (total = 79.5%).

Table 4 presents the results disaggregated by type of problem. The total percentage reporting significant between-group or within-group differences favoring PS ranged from 66.7% for Addiction (Drug, Alcohol, Cigarette Smoking) to 90.0% (for prevention and management of cardiovascular disease). The median of these is 83.3%.

**Table 4:** Results of studies disaggregated by type of health problem addressed

Results by Type of Health Problem Addressed	Significant between-group	Significant within group	Total favoring peer support
Addiction: Drug, Alcohol, Cigarette Smoking	2 (66.7%)	0	2/3 (66.7%)
Cardiovascular disease, including Heart Failure and general prevention through any or all of diet, exercise, PA, BP mgmgt	4 (40.0%)	5 (50.0%)	9/10 (90.0%)
Diabetes, including support for parents of children with diabetes	6 (66.7%)	2 (22.2%)	8/9 (88.9%)
HIV/AIDS	3 (50.0%)	2 (33.3%)	5/6 (83.3%)
Maternal & Child Health: Pregnancy, Childbirth, Pre- and Post-natal care, including breastfeeding	14 (82.4%)	1 (5.9%)	15/17 (88.3%)
Mental Health, including post-partum depression (3)	5 (62.5%)	1 (12.5%)	6/8 (75.0%)
Other Chronic Disease	6 (50.0%)	3 (25.0%)	9/12 (75.0%)
Totals	39 (60.0%)	14 (21.5%)	53 (81.2%)

#### Review of reviews

Table 5 summarizes the 24 review studies identified in this search. Five of the reviews presented overall results in a format that did not allow detailed quantification of their results (Campbell 2004, Chang 2010, Nemcek 2003, Parry 2010, Postma 2009) [5, 47–50]. (For convenience of reader, papers are referred to in the text by first author and year but are included in references as per the reference number.) For the remaining 19, it was possible to disaggregate the findings of individual reviews according to specific subtopics, e.g., knowledge changes, behavioral changes, clinical improvement, quality of life. For each of these 19, then, Table 5 presents percentage of papers finding effects in each subcategory. It also presents the range and median of these percentages for each review. For example, the review of antenatal support for breastfeeding by Ingram et al. [51] disaggregated results for (a) all women regardless of interest in breastfeeding, and (b) women considering breastfeeding. For the former group, 1 of 4 RCTs reported a significant effect (25%), and 3 of 3 non-RCTs reported a significant effect (100%). For the group of studies among women considering breastfeeding, 2 of 3 RCTs reported a significant effect (67%) and 1 of 1 non-RCT reported a significant effect (100%). For the whole review of Ingram et al., the percentages reporting significant effects were then 25%, 100%, 67% and 100% so Table 5 reports the range of these as 25% to 100% with a median of 67%. These data are then summarized in Table 6. Across all 19 reviews, the lower end of the range of percentage of papers reporting an effect varied from 0 to 90% with a mean of 36% and median of 40%. The upper end of the range of percentage of papers reporting an effect varied from 50 to 100% with a mean of 85.2% and median of 89%. The median of the medians for the 19 papers was 64.5%.

**Table 5:** Summaries of reviews of peer support interventions

Title (author, year) number of studies	Topic	Peer support interventions peer support defined as	Authors’ conclusions [comments of present authors]	Range (median) of effects reported
Effect of antenatal peer support on breastfeeding initiation: a systematic review (Ingram et al., 2010) [51] 11 studies	Breast Feeding	Antenatal PS to promote initiating breastfeeding. PS “offered by women who had themselves breastfed, who were usually from the same socioeconomic background and locality as the women they were supporting and who had received appropriate training.” (p. 1740)	For all women regardless of interest in breastfeeding: 4 RCTs – no significant pooled effect; 1/4 showed significance (25%). 3/3 nonRCTs (100%) showed effect. For women considering breastfeeding: 3 RCTs – pooled effect significant (*p* = 0.04); 2/3 showed significance (67%). 1 nonRCT reported significant effect.	25%–100% (67%)
Outcome effectiveness of the lay health advisor model among Latinos in the United States: an examination by role. (Ayala et al., 2010) [108] 17 studies	Varied	Evaluated two roles: Educator Only – “usually involved several home visits and/or group classes” and Educator plus Bridge to other services – “generally consisted of one or two individual contacts in a participant’s home or at the clinic” (p. 827).	Among Educator Only, 5 of 6 (83%) showed effect for health behaviors and 3 of 6 (50%) showed effects in health status Among Educator plus Bridge, 10 of 11 (91%) reported effects in health behaviors, utilization, and/or clinical status.	50%–91% (83%)
A review of the literature on peer support in mental health services. (Repper & Carter, 2011) [109] 40 studies	Mental Health	Varied roles in mutual support, consumer-run services, and providing support as part of broader services, varying from a “reciprocal relationship to a less symmetrical relationship of ‘giver’ and ‘receiver’ of care” (395).	5 RCTs examining effects on Sx, utilization, social functioning, etc.: 2 showed effects of PS (40%) 9 other quantitative studies of effects on utilization, social functioning, etc.: 8 (89%) showed effect of PS	40%–89% (64.5%)
Can community health workers improve adherence to highly active antiretroviral therapy in the USA? A review of the literature. (Kenya et al. 2011) [110] 16 studies	HIV/AIDS	Delivery of culturally appropriate health education, assistance with accessing services, provision of direct services (e.g., medication administration), medication reminders, accompaniment to apptmts. PS personalized based on individual needs and socio-environmental determinants (p. 526) Many employed Directly Observed Therapy (DOT)	In 13/16 (81%) studies, “CHW model contributed to measurable HIV viral load suppression and/or improved CD4 cell count.” (p. 527). 7/16 (44%) reported significant findings. 12/13 successful interventions used DOT. 2/3 studies that did not find evidence for PS compared PS to “alternative HAART adherence interventions” “Interventions that lasted at least 24 weeks, provided frequent contact …, and focused on medication management were associated with improved” adherence (Abstract).	44%–81% (62.5%)
An integrative review of community health workers in type 2 diabetes. (Hunt et al., 2011) [97] 16 studies	Diabetes	Support, counseling, education, case management, advocacy, program facilitation, coordinating and conducting educational programs and courses, linking patients and professionals, leading peer support meetings	RCT or controlled designs: 4/5 (80%) reported significant between group tests of program effects Within-Group, Pre-Post designs: 8/8 (100%) reported effects	80% - 100% (90%)
Breastfeeding peer counseling: From efficacy through scale-up (Chapman et al., 2010) [111] 26 studies	Breastfeeding	Studies classified: low-intensity – only prenatal education, or if postpartum contact primarily by telephone: high-intensity – ≥3 contacts, both prenatal and postpartum support, most contacts in person.	Initiation of Breast Feeding: 3/4 high intensity, 0/3 low intensity Duration of Breast Feeding: 5/9 high intensity, 1/5 low intensity Exclusivity of Breast Feeding: 2/5 studies designed to promote breast feeding but not necessarily exclusive breast feeding 7/7 designed to promote exclusivity Significant reductions in diarrhea in 4 of 5 studies.	0% - 75% (37.5%) 20% - 56% (38%) 40%–100% (70%) 80%
Effectiveness of community health workers in Brazil: A systematic review. (Giugliani et al. 2011) [112] 23 studies	Maternal and Child Health	In Brazil, 240,000 Community Health Agents (staff as part of health system’s primary care teams) serve 118 million citizens. Additionally, Community Health Workers work as volunteers such as in church-based programs.	For categories addressed by at least 4 studies, numbers and %s of studies finding positive results: frequency of weighing children - 4/4, 100%; attend prenatal care – 4/6, 67%; immunizations – 4/5, 80%; breastfeeding – 4/5, 80%; use of oral rehydration for diarrhea – 4/7, 57%; knowledge of oral rehydration – 4/6, 67%; stunting – 0/4, 0%.	0% - 100% (67%)
Lay health workers providing primary care for maternal and child health. (Lewin et al., 2010) [113] 82 studies	Maternal and Child Health	Cochrane Collaborative review of lay health workers, “paid or voluntary…who: performed functions related to healthcare delivery, was trained in some way in the context of the intervention, but had received no formal professional or paraprofessional certificate or tertiary education degree.” (p. 7)	Numbers of RCTs (%, RR for effect when significant) reporting significant effects: immunizations – 3/6, 50%, 1.23; mortality under 5 years – 0/3; neonatal mortality – 0/4; reported childhood illness – 0/7; care seeking – 1/3, 33%; initiated breastfeeding – 6/12, 50%, 1.36; any breastfeeding – 5/12, 41.7%, 1.24; exclusive breastfeeding – 7/10, 70%, 2.78; cure for TB – 1/4, 25%, 1.22; cure for new TB – 1/2, 50%; cure and completed trtmt for TB – 1/3, 33%; completed Isoniazid trtmt for TB prev – 0/3	0% - 70% (33%)
The effect of peer support programs on depression. (Pfeiffer et al., 2011) [114] 7 studies	Depression	Regular contact with at least one other person with depression. Groups could be professionally led, however needed to … be described as peer support (or mutual support or self-help) or to be organized so participants determined majority of topics, content of discussion. Included varied formats, e.g., group, pairs, telephone. CBT conditions were group delivered.	Pooled standardized mean difference, PS vs UC = **−** 0.59, *p* = 0.002. 5/7 studies showed significant differences favoring PS. Pooled standardized mean difference, PS vs group CBT = 0.10, NS. 0/8 studies showed significant differences favoring PS. NB: showed PS equal to group CBT.	0% - 71% (35.5%)
Evaluating outcomes of CHW programs. (Viswanathan et al., 2010) [2] 53 studies	Varied	Performs health-related tasks beyond peer counseling or peer support alone to create bridge between community members, especially hard-to-reach populations, and health care system. Health training associated with the intervention shorter than professional worker, not part of a tertiary education certificate. Recognized or identified as member of the community in which works (p. 793).	Outcomes in specific areas: Knowledge – Moderate evidence in 2/3 areas (67%) Health Behavior – Moderate evidence in 3 of 21 areas (14%) Health Care Utilization – Moderate evidence in 4 of 12 areas (33%) Costs & Cost-Effectiveness – Moderate evidence in 1 of 4 (25%)	14% - 67% (29%)
Peer support telephone calls for improving health. (Dale et al., 2008) [115] 7 studies	Varied	Telephone calls (of any duration) in which the peer has similar or relevant health experience (p. 4).	Increases in mammography, maintained mammography, healthy diet in post-MI patients, continuation of breastfeeding, reduced Sx of post-partum depression.Numbers of studies (%) finding significant effects: Physical Health Outcomes: 0/3 (0%) Psychological Health Outcomes: 2/5 (40%) Self Efficacy: 0/2 (0%) Mental Health: 2/2 (100%) Quality of Life: 0/1 (0%) Satisfaction with Intervention: 1/2 (50%) Health Behaviors: 4/5 (80%)	0% - 100% (40%)
Systematic review of peer-support programs for people with cancer (Hoey et al., 2008) [83] 43 studies	Cancer	Peers provided support to people with cancer; peer had been diagnosed and/or treated for cancer; primary purpose of the program was to provide support to cancer patients (p. 316).	Mostly qualitative findings: “high level of satisfaction” and/or indicators of acceptance. Of 8 RCTs, 3 (37.5%) reported effects. Of 4 nonRCT studies w/ quantified findings, 2 (50%) reported effects.	38% - 50% (44%)
Effectiveness of community health workers programs for hypertension. (Brownstein et al., 2007) [116] 14 studies	Hypertension	Health education re: behavioral risks, changes in lifestyle, adherence, barrier reduction, facilitate services (e.g., insurance), instrumental support (e.g., transportation for care), measuring and monitoring blood pressure, social and emotional support, and mediation with health care and social services.	Numbers of studies (%) finding effects: Behavioral changes: 9/10 (90%) Adherence: 5/5 (100%) Blood pressure: 9/10 (90%)	90%–100% (90%)
Systematic Review of U.S.-Based Randomized Controlled Trials Using Community Health Workers (Gibbons & Tyus, 2007) [3] 12 studies	Varied	“community members who serve as connectors between health care consumers and providers to promote health among [those traditionally lacking] … adequate access to care” (p. 371). Included home visits, educational sessions, distribution of health education materials, personalized counseling.	Overall, 10/12 (83%) RCTs “demonstrated … efficacy in enhancing outcomes” (abstract). Numbers of RCTs (%) reporting effects in categories indicated: Mammography: 3/3 (100%) Cervical cancer screening: 3/5 (60%) Other areas (enrollment in research, early intervention for developmental disabilities, healthy diet, blood pressure, maternal and child health): 4/6 (67%)	60%–100% (75%)
Community health worker programs for diabetes management. (Norris et al., 2006) [117] 18 studies	Diabetes	Any healthcare worker who: (i) carried out functions related to healthcare delivery; (ii) trained in some way in the context of the interventions; (iii) no formal professional or paraprofessional training in healthcare; and (iv) had relationship with the community served” (p. 545).	Numbers of studies (%) reporting effects in categories indicated: Knowledge of diabetes/self care: 5/7 (71%); Blood glucose (Hemoglobin A1c): 4/11 (36%); Lipids: 2/5 (40%); Blood pressure: 2/4 (50%); Emergency and/or hospital care: 3/4 (75%)	36%–75% (50%)
Social support interventions for diabetes. (van Dam et al., 2005) [118] 6 studies	Diabetes	Variety including group medical visits, peer group, peer internet, inclusion of spouse, family of friends in intervention.	Numbers of studies (%) reporting effects in specific categories: Knowledge of diabetes: 2/3 (67%); Self management: 3/3 (100%); Psychosocial factors, QOL: 4/5 (80%); Clinical, biomedical (e.g., HbA1c): 2/5 (40%)	40%–100% (73.5)
Use of community health workers in research with ethnic minority women. (Andrews et al., 2004) [119]. 24 studies	Varied	Varied roles: educator – 18 studies, outreacher – 14 studies, case manager – 4 studies, data collector, e.g., Pap tests, breast exams in remote villages – 1 study.	Qualitative, descriptive, quasi-experimental findings: effective in increasing access to health services, knowledge and behavior change among ethnic minority women (abstract). Numbers of studies (%) reporting effects in specific categories: Knowledge: 4/6 (67%) Behavior: 7/9 (78%) Access: 14/14 (100%)	67%–100% (78%)
Health related virtual communities and electronic support groups: Systematic review of the effects of online peer to peer interactions. (Eysenbach et al., 2004) [21] 38 studies	Varied	“virtual community” defined as individuals with similar health related interests and predominantly nonprofessional backgrounds who interact and communicate publicly through a computer communication network (p. 1167).	From 38 studies, identified 6 RCTs: 4 reported effects (67%) 2/3 evaluating impacts on depression reported effects (67%) 1/2 evaluating impacts on diabetes reported effect (50%)	50%–67% (68.5%)
Outcome evaluations of CHW programs. (Swider, 2002) [1]. 20 studies	Varied	Identified papers using terms “community health worker,” “community health advocate,” *“promotora de salud,”* “community health promoter,” “lay health worker,” and “community outreach worker.”	Numbers (%) reporting effects of PS in categories indicated: Health status: 3/4 (75%) Behavior change: 5/6 (83%) Cost: 1/2 (50%)	50%–83% (75%)
Peer support programs for cancer. (Campbell et al., 2004) [47]. 18 studies	Cancer	One-to-one, group, telephone and internet support programs, some with professional facilitation.	Across varied designs, “consistent informational, emotional and instrumental effects were identified” (abstract). However, 3 RCTs evaluating peer-led support groups found mixed and/or negative results on QOL; see text Discussion of Possible Harm of Unmoderated PS.	0/3 (0%)
Indigenous healthcare worker involvement for indigenous adults and children with asthma (Chang et al. 2010) [48] 1 paper	Asthma	Review of Indigenous Health Workers “Indidgenous” as “group of people who have inhabited a country for thousands of years, which often contrast with those of other groups of people who reside in the same country for a few hundred years” (p. 3), e.g., Australian Aboriginal, First Nations, Native Hawaiian.	Found only one study with children with asthma meeting Cochrane Collaboration criteria. Significant difference on asthma knowledge favored group with Indigenous Health Worker, but “although not statistically significant, all the outcomes favoured the group that had IHW involvement in the asthma education program” (Abstract)	Not Applicable
Review of CHW evaluations. (Nemcek & Sabatier, 2003) [49] 10 studies	Varied	Outreach, culturally sensitive care, health education/counseling, advocacy, home visits, health promotion/lifestyle change, transportation/homemaking	Identified 18 studies through 10 papers. 11 of 18 assessed outcomes, each of which reported ≥1 effect of PS.	100%
Peer support intervention trials for individuals with heart disease: A systematic review (Parry & Watt-Watson, 2010) [5] 6 studies	Heart disease	“peer mentors,” “lay health workers,” and “peer informants” delivered one-to-one sessions, telephone calls, combination of one-to-one and telephone calls, or self-help/support groups.	Some evidence for effects but authors indicated methodological problems preclude generalizations. Three of 6 studies reported some effect for peer support.	50% (50%)
Community health workers and environmental interventions for children with asthma. (Postma et al. 2009) [50] 7 studies	Asthma	PS worked in homes with families to promote behaviors that would reduce environmental triggers for asthma (e.g., controlling exposure to cockroach, dust mite, cigarette smoke).	From abstract: “Overall, the studies consistently identified positive outcomes associated with CHW-delivered interventions, including decreased asthma symptoms, daytime activity limitations, and emergency and urgent care use” (p. 564) From results provided, not possible to characterize significance of PS vs control comparisons	Not Applicable

**Table 6:** Statistical summary of 19 previously published reviews of peer support for which it was possible to abstract percentages of studies reporting effect in specific categories of application of peer support

			Lowest, highest, and median %s of studies identified as reporting effects in specific categories of application		
First author	# studies	Year	Lowest	Highest	Median
Ingram	11	2010	25	100	67
Ayala	17	2010	50	91	83
Repper	40	2011	40	89	64.5
Kenya	16	2011	44	81	62.5
Hunt	16	2011	80	100	90
Chapman	26	2010	0	75	37.5
Gugliani	23	2011	0	100	67
Lewin	82	2010	0	70	33
Pfeiffer	7	2010	0	71	35.5
Viswanathan	53	2010	14	67	29
Dale	7	2008	0	100	40
Hoey	43	2008	38	50	44
Brownstein	14	2007	90	100	90
Gibbons	12	2007	60	100	75
Norris	18	2006	36	75	50
van Dam	6	2005	40	100	73.5
Andrews	24	2004	67	100	78
Eysenbach	38	2004	50	67	58.5
Swider	20	2002	50	83	75
Means			36	85.2	60.7
Medians			40	89	64.5

### Extended review of peer support in diabetes management

#### Study selection

From the broad review through July 15, 2011, the nine studies addressing diabetes prevention (2 studies) and management (7 studies) were retained. Using the same syntax as for the broader review with the addition of the key word, “diabetes” and searching for dates July, 2011 through December 31, 2015 identified an additional 33 studies. Using the same inclusion and exclusion criteria as for the broader review, seven were excluded because the intervention was provided by a professional, one because the intervention was delivered by both a nonprofessional and a professional, confounding the effect of peer support, three because the intervention was limited to a fixed number of group programs or classes or peers implementing a highly scripted information program or care coordination with limited peer support, and two because the study represented a secondary analysis of a study already included. One paper [52] reported on two distinct interventions so was treated as two studies. That left a total of 30 studies in the review, nine from the original, broader review and 21 identified through the review of diabetes studies through December 31, 2015.

#### Study characteristics

Details of all 30 studies are described in Additional file 2, “Details of Studies Included in Review of Peer Support in Diabetes Management.”

The 30 studies in the final sample represented nine countries: 19 from the United States, three from England, two from China, and one from each of Argentina, Cameroon, Guatemala, Ireland, Netherlands, and New Zealand. Twenty five were from World Bank designated high-income countries and five from low-income, low-middle, and high-middle-income countries. Two focused on prevention and 28 on management.

As detailed in Table 7, 22 of the studies used randomized controlled designs (RCTs) (including cluster randomized designs), three used other controlled designs (such as multiple baseline), five used within-groups, pre-post designs, and one used a comparison group of age- and sex-matched controls [53].

**Table 7:** Outcomes of peer support interventions for diabetes management and prevention disaggregated by type of design and type of measure. (Percentages within each category of design and measure)

	Randomized, controlled trials		Other controlled designs		Within-group, pre-post designs		Other designs		Totals
	Objective	Standardized	Objective	Standardized	Objective	Standardized	Objective	Standardized	
Significant between groups	8 (61.5)	5 (62.5)	3 (100)	0	NA	NA	1 (100)	0	17 (56.7)
Significant within groups	1 (7.7)	3 (37.5)	0	0	4 (100)	1 (100)	0	0	9 (30.0)
Nonsignificant	4 (30.1)	0	0	0	0	0	0	0	4 (13.3)
Counter	0	0	0	0	0	0	0	0	0
Totals	13	8	3	0	4	1	1	0	30

#### Synthesis of results

Table 7 presents the results of the 30 diabetes studies disaggregated by type of design, type of outcome measure, and outcome. Across all 30 studies included in this review, 17 (56.7%) reported significant, between-group differences favoring PS. An additional 9 (30.0%) reported significant within-group changes indicating effects of PS among those who received it. Together then, 26 of 30 studies (86.7%) reported significant effects of PS. Four studies (13.3%) reported no significant effects of PS, and no studies reported results counter to effects of PS.

Aggregating all 21 RCTs in Table 7, a total of 13 (61.9%) reported significant between-group differences, another 4 (19.1%) reported significant within group differences, so that a total of 17 of the 21 (81.0%) reported significant between- or within-group differences favoring PS. Disaggregating by type of measure, 8 of 13 RCTs that used objective measures reported significant between-group differences and another one of these 13 reported significant within-group differences. Thus, a total of 9 of 13 (69.2%) RCTs using objective measures reported significant between- or within-group differences indicating effects of PS. Adding in those using standardized measures, a total of 17 of 21 (81.0%) RCTs using objective or standardized measures reported significant between-group (13 of 21, 61.9%) or within-group (4 of 21, 19.1%) differences indicating effect of PS.

Table 8 presents the data aggregating across the 24 studies utilizing RCT or Other Controlled Designs. Aggregating also across both Objective and Standardized measures, 16 of 24 (66.7%) of studies reported significant between-condition effects favoring PS, and an additional 4 (16.7%) reported significant within-condition effects. Combining these, 20 of 24 or 83.3% of RCT or Other Controlled Designs using objective or standardized measures reported significant effects of PS with diabetes.

**Table 8:** Outcomes of randomized controlled trial and other controlled design evaluations of peer support interventions in diabetes prevention and management by type of measure. (Percentages within each category of design and measure)

Outcome	Type of measure		Totals
	Objective	Standardized	
Significant between groups	11 (68.8)	5 (62.5)	16 (66.7)
Significant within groups	1 (6.3)	3 (37.5)	4 (16.7)
Nonsignificant	4 (25.0)	0	4 (16.7)
Counter	0	0	0
Totals	16	8	24

Among the 30 diabetes studies, 19 reported pre- and post-intervention values of HbA1c among those receiving PS [52–69] and another four reported average changes from pre- to post-intervention [70–73]. Across all 23, the average change score was a decrease of 0.76 (*p* < 0.001). Among the 19 reporting pre- and post-values, repeated measures analysis controlled for the number of participants in the PS condition and the duration from pre-intervention to post-intervention/follow-up appointment. The average HbA1c declined from 8.42 to 7.63% (*p* = 0.004) with an average of 140 participants in PS condition (range = 14 to 781) followed an average of 12.21 months (range = 4 to 24). Neither the number of participants or duration of intervention and follow-up significantly influenced the change in HbA1c over time.

## Discussion

### Summary of evidence

Across all 65 investigations included in the initial review, 54 or 83.1% found evidence of effects of PS. These include 31 between-group findings favoring PS out of 43 (72.1%) RCT or other controlled trials using objective or standardized outcomes and another 5 (11.6%) reporting significant within group changes. Thus, among RCTs and other controlled trials using objective or standardized measures 83.7% showed significant effects of PS. Within the several categories of design and type of outcome measure, the percentages showing effects of PS ranged from 71.4 to 100%. Similarly, across types of health problems, the percentage of studies reporting significant results for PS ranged from 66.7% for Addictions (Drug, Alcohol, Cigarette Smoking) to 90% for prevention/management of cardiovascular disease. Among 24 review papers, 19 provided quantifiable findings including a median of 64.5% of papers across individual reviews reporting effects of PS. Finally, among the 30 studies identified through December, 2015 that evaluated peer support with diabetes, 86.7% reported significant between or within group differences for peer support. Of the 24 diabetes studies using objective or standardized measures in RCT or Other Controlled Designs, 83.3% reported significant between or within group differences for peer support. The average reduction in HbA1c was 0.76 points among those 23 reporting such results. The evidence for PS is broad.

### Analysis of interventions with nonsignificant effects

Table 9 includes details of the nine studies that reported null and two that reported negative findings in the original review and four additional studies that reported null findings in the extended review of diabetes. For ease of reference, these are indicated in the table and text by last name of first author and year of publication. As detailed in the notes in Table 9, all 15 fall into one or more of the following five categories:

1. Other or Competing Sources of Social Support -- PS was tested as addition to interventions that already included appreciable social support (Hunkeler 2000; May 2006; Muirhead 2006) [38, 74, 75]
2. Implementation Problems -- problems with implementation of PS intervention (Muirhead 2006) [75] including failure to implement with all for whom the PS was intended (Chen 2010) [42].
3. Methodological Problems including non-equivalent controls and contamination across conditions (Chen 2010) [42] or substantial support within control/usual care conditions including quarterly mailings and phone calls (Palmas, 2014) [76] and “standardized care” that included quarterly feedback and suggestions for improved management for both primary care providers and patients (Chan, 2014) [70].
4. Lack of Acceptance of PS Intervention (Graffy 2004; Hunkeler 2000; May 2006; Murihead 2006; Simoni 2007; Simmons, 2015; Smith 2011; Vilhauer 2010; Palmas 2014) [38, 58, 67, 74–79], discussed further below.
5. Possible Harm of Unmoderated PS (Kaplan 2011, Nicholas 2007, Salzer 2010, Vilhauer 2010) [79–82], discussed further below.
6. Lack of overall effects but significant effects within subgroups (Chan 2014, Simmons 2015) [67, 70], also discussed further below.

**Table 9:** Details of studies reporting negative or null findings for effects of peer support

Description and citation	Design	Results	Possible explanations of null findings of findings counter to peer support
Chan et al., 2014 [70] Patients with well controlled diabetes trained to provide telephone support for others with diabetes. Protocol called for 12 calls over 1 year	RCT of standardized care versus standardized care plus peer support.	No significant differences between groups on clinical measures	Standardized care provided high quality clinical care including initial reports on medication adherence, self management, recommendations for physicians and patients; periodic status updates and recommendations to patients [90] Subgroup Effects As noted in text, among approximately 20% above norms for distress, the combination of peer support and standardized care substantially reduced distress and hospitalizations.
Chen et al., 2010 [42] Health coaches (medical assistants or health workers) paired with 1st-year residents in language concordant, stable teamlets for patients with hypertension and/or diabetes	Compared to usual care in teams of 2nd- and 3rd-year residents	Improvements in intended process measures (e.g., assessment of LDL, BMI, smoking; setting self-mgmt plan) but not patient clinical indicators (e.g., HbA1c, BP)	Implementation Problems Not all received health coaching that was applied according to “time and prioritization of patients who were more complicated or needed more assistance” (p. S611). Methodological Problems Study designed to show feasibility within clinical setting rather than outcomes. Non-equivalent controls. Potential contamination: (i) some 2nd and 3rd year residents having participated in pilot testing of health coaching and/or training of 1st year residents in chronic care, (ii) overlap in attendings for 1st and 2nd/3rd year residents, and (iii) nursing staff who provided health coaching also interacting “regularly with all clinic patients as medical assistants and health workers” (p. S613).
Graffy, 2004 [77] Intervention to increase breastfeeding compared 1) PS and postnatal in-person visits plus phone calls on request vs 2) UC	Individual randomized design among sample of 720 from among 844 eligible mothers.	No differences in self reported % breast feeding initially or at 4 or 6 mos post-partum	Lack of Acceptance Although initial antenatal contact by PS counselors achieved in 80% of those randomized to receive it, post-natal contact only if initiated by mothers and occurred for only 62% (p. 3)
Hunkeler et al. 2000 [38] Augmentation of antidepressant therapy compared 1) Nurse telehealth care including medical and emotional support and advice over the phone plus PS involving in-person support and telephone calls by peers recovered from depression to 2) Nurse telehealth alone, to 3) UC	RCT compared Nurse telehealth + PS to Nurse telehealth alone and UC among 302 drawn from 370 eligibles; 68 refused informed consent.	No differences reported on Hamilton Depression Rating Scale or Beck Depression Index or on SF-12 Mental & Physical Composite Scales at 6-week or 6-month follow-up.	Other Sources of Support Control included Nurse telehealth care including medical and emotional support and advice Lack of Acceptance: Of 62 Randomized to Nurse telehealth + PS, 31 (50%) had one or fewer contacts among whom 20 (32.3%) had no contacts – refused (11) or no contact (9). 13 had 2 contacts, 14 had 3–5, 4 had 9–20. Only 6 had at least 1 face-to-face
Kaplan, 2011 [80] Tested unmoderated, unstructured Internet peer support for individuals with serious mental illness.	RCT compared Internet peer support via listserv, via bulletin board, or control. 300 with Schizophrenia Spectrum or Affective Disorder	No differences on measures of recovery, quality of life, empowerment, social support, or distress	Possible Harm of Unmoderated PS Unmoderated listservs may be inappropriate for those with serious mental illness (as in present case) or other highly stressful diseases or conditions.
May, 2006 [74] Group-based smoking cessation plus support from another group member in person and by telephone vs. group treatment without group member support	630 randomized to 34 grps (14 with peer support, 20 without). 96 excluded for failure to attend visit 2 quit date.	1-week post quit, borderline (*p* = 0.06) difference in abstinence (need to check) favored peer support (OR = 1.45, 0.92–2.29). No other differences between groups.	Other Sources of Support All participants, including controls, participated in group program for smoking cessation. Lack of Acceptance Peer support appears to have been first introduced in visit 2 quit date and moderately embraced by participants: mean = 2.7 phone calls in first week after quit date, dropped to 1.2, 1.1 and 0.7 in following weeks. (p. 240)
Muirhead, 2006 [75] “Normal breastfeeding support” (involving a community midwife for 10 days, breastfeeding support groups and breastfeeding workshops) vs Normal breastfeeding support plus the assistance of two peer supporters	Of 284 pregnant women recruited through a physician practice, 59 declined and 225 were randomized to conditions.	No differences in self-reported breastfeeding initiation or duration at 10 days, 8 weeks or 16 weeks post-partum.	Other Sources of Support Hospital midwives helped mothers in both groups to initiate breastfeeding. With extensive support in “normal breastfeeding support,” peer support challenged to add additional influence. Implementation Problems Peer supporters had little/no contact in hospital but were available to women after returning home and if peer supporters were informed in time. Mothers still breastfeeding on return home contacted by peer supporters every 2 days or as often as required either by phone or a personal visit up until day 28. If requested by the mother, the same peer supporters provided further support after 28 days until 16 weeks. (pp. 193–194) No data on actual contacts. “…Women in the peer support group who did not commence breastfeeding or who stopped while still in hospital received no peer support postnatally” but were included in intention-to-treat analyses of outcomes (p. 196). Lack of Acceptance “…half of the women in the population simply did not want to breastfeed....” Lack of acceptance among professionals: “The support and cooperation of health professionals is required for peer supporters to function, and some may be unwilling to accept lay people being involved in the care of women” (p. 196). Methodological Problems Possible social desirability bias of outcomes in that 10-day assessment surveys completed in presence of health visitor and both 8- and 16-week assessments completed in presence of physician or practice nurse. (p. 194)
Nicholas, 2007 [81] 34 family caregivers of technology-assisted children with chronic lung disease were recruited from a patient database and assigned to dyads for information sharing and support.	Non-randomized and no comparison group.	No significant within group differences over time for perceived social support from friends or family, caregiver stress, coping or social isolation (Meaning of Illness Questionnaire, Coping Health Inventory for Parents	Possible Harm of Unmoderated PS Family caregivers were already under substantial stress. May have been unrealistic to expect them to support each other as opposed to receiving support from a trained supporter. Dyads may fit into pattern of lack of effect for unmoderated support among those with highly stressful diseases or conditions. Paper did identify effects through qualitative study
Palmas 2014 [76] For adults with diabetes and elevated HbA1c (> 8%), 12-month CHW intervention included one-on-one visits, group visits, and telephone follow-up.	RCT of PS versus enhanced usual care	No significant differences between groups on clinical outcome measures.	Lack of Acceptance “…in over half of the intervention group, the CHWs were not able to deliver any of the planned one-on-one or small group sessions and only able to contact participants by phone” (p. 968). Adjustment for number of contacts led to a borderline (*p* = 0.054) effect for the CHW intervention (p. 967).
Salzer, 2010 [82] Unmoderated internet peer support listserv for women recently diagnosed with breast cancer	Random assignment to peer support listserv or Internet-based educational control condition Data collection at baseline, 4 and 12 months.	Control group showed significantly greater effect on FACT-B (4 and 12 months, ps < .05). No significant differences between groups on MOS Social Support Scale.	Possible Harm of Unmoderated PS Unmoderated listservs may be inappropriate for those with highly stressful physical illness like newly diagnosed cancer (as in present case) or those with other highly stressful diseases or conditions
Simoni, 2007 [78] Appraisal, spiritual, emotional, and informational adherence-related support vs. UC to improve antiretroviral medication adherence and depressive symptomatology in HIV+ men and women. Support = 6, semi-monthly group meetings and weekly contact by peer supporters over 3 mos.	136 participants enrolled (71 randomized to peer intervention and 65 to UC). 53% of eligible patients approached declined to participate… [due to] lacking interest, being too busy, transportation difficulties... or being asocial.” (p. 491)	No significant within or between group differences for adherence based on Electronic Drug Monitoring. Relationship between attendance and lower deppressive Sx at 6 mos	Lack of Acceptance Those assigned to support condition attended average of 2.1 of 6 meetings. 23% attended none, 26% attended 2, and only 17% attended 5 or 6 of 6 peer meetings. (p. 491) Average number telephone contacts for intervention participants was 5.8 (Range = 0 to 17). (p. 492) Concern for confidentiality may have suppressed participation in group sessions.
Simmons et al. 2015 [67] Support by trained patients with diabetes in individual, group, or individual plus group modes over 8–12 months	2 × 2 factorial randomised cluster design of individual peer support, group peer support, individual plus group, or usual care	No significant differences between peer support versus usual care in changes on clinical indicators.	Lack of Acceptance “only 61.4% (592/977) of intervention participants attended an actual peer support session.” Implementation may have been compromised by scope: 127 peer support facilitators in group, individual, and combined group and individual arms, 2 × 2 factorial, cluster randomized design with 1299 randomized participants drawn from three counties and “… 62 general practices, a hospital clinic and Diabetes UK members.” One nurse served as the principal study manager. Subgroup Effects Significant differences favoring group or group plus individual for systolic blood pressure.
Smith et al. 2011 [58] Peer led groups including presentation and discussion of topics in diabetes management. 9 sessions scheduled over 2 years	Cluster randomized design. Practices assigned to peer-leg groups or standard care	No significant between-group differences in primary (HbA1c blood pressure, cholesterol) or secondary (BMI) outcomes.	Lack of Acceptance Mean of 5 of 9 sessions attended; 18% attended 0 Questionable whether peer support provided, e.g., infrequent meetings (9 over 24 months) that appear to have been focused on discussion of topics in diabetes management; participants discouraged from contacting peer leaders between meetings
Vilhauer, 2010 [79] Unmoderated online support among women with metastatic breast cancer. Women sent an introductory email with instructions on how to access group and basic ettiquette.	From over >900 mailings and phone calls to oncologists, breast cancer clinics, and support centers, 42 women replied and 31 determined eligible. Nonrandom assignment to three online support groups to restrict group membership to 10 or 11. Compared to wait-list control.	Among controls, significant within group differences in breast cancer related distress (FACT-B breast cancer subscale, *p* < .04) and in daily activity (ECOG, *p* < .04) over first month. At 2 months, control group reported higher activity scores (ECOG, *p* = .02).	Lack of Acceptance 31 from over 900 mailings engaged in online resource.However, 73% retention rate and average participation of 5.69 days/wk. Average 82 min spent reading messages per week and average 69 min spent writing messages per week. Possible Harm of Unmoderated PS Unmoderated listservs may be inappropriate for those with highly stressful physical illness like metastatic breast cancer (as in present case) or with other highly stressful diseases or conditions

Lack of acceptance of the PS intervention on the part of those for whom it was intended appears to have been a feature of nine of the studies (Graffy 2004; Hunkeler 2000; May 2006; Muirhead 2006; Simoni 2007; Smith 2011; Vilhauer 2010; Palmas 2015; Simmons 2015) [38, 58, 59, 67, 74, 75, 77–79] Two of these (Hunkeler, 2000; May 2006) [38, 74] added PS to interventions which already offered appreciable support. A third study among HIV-positive adults suggests that concerns for confidentiality may limit acceptance of PS (Simoni, 2007) [78]. In a fourth, lack of acceptance of the goals of the study may have accounted for the poor acceptance of PS for breastfeeding when offered to women among whom half “…simply did not want to breastfeed…” (Muirhead 2006, p. 196) [75]. This may have been exacerbated by lack of acceptance of PS by professionals in the setting among whom the authors speculated “…some may be unwilling to accept lay people being involved in the care of women” (p. 196). Failure with those who are not interested in breastfeeding was also noted in the review of Ingram, 2010. [51] A fifth (Vilhauer 2010) [79] appears to have demonstrated that mailings announcing the availability of an online support resource are not effective; 900 mailings yielded only 31 participants.

Five of the nine studies illustrating Lack of Acceptance raise the issues of frequency of contact and how PS is presented and promoted. In Smith 2011 [58], PS classes were offered less than bimonthly (9 over 24 months) and participants were discouraged from contacting the peer supporters between meetings. Instead of using peers to engage those who did not attend, project staff contacted them. Additionally, the meetings appear to have focused more on discussion of a series of topics than on exchange among participants or their individualized goals for self management. In Graffy 2004 [77], PS for breastfeeding after the mother returned home from hospital was left to be initiated by the mothers. Similarly, in Muirhead 2006 “…Women in the PS group who did not commence breastfeeding or who stopped while still in hospital received no PS postnatally….”(p. 196) [75]. In the Palmas 2014 study, “…in over half of the intervention group, the CHWs were not able to deliver any of the planned one-on-one or small group sessions and only able to contact participants by phone” (p. 968). Extending this line of reasoning, adjustment for number of contacts led to a borderline (*p* = 0.054) effect for the CHW intervention (p. 967) [76].

The study of Simmons and colleagues [67] perhaps points to challenges of scope and complexity that may explain low participation. It deployed 127 peer support facilitators in group, individual, and combined group and individual arms of a 2 X 2 factorial, cluster randomized design with 1299 randomized participants drawn from three counties and “… 62 general practices, a hospital clinic and Diabetes UK members.” One nurse served as the principal study manager. “…Only 61.4% (592/977) of intervention participants attended an actual peer support session.”

Possible harm of unmoderated PS emerged in several interventions for individuals beset by substantial health problems or stress. This includes serious mental illness (schizophrenia spectrum or affective disorder, Kaplan, 2011) [80] or cancer, as in the papers reporting interventions for those with newly diagnosed (Salzer, 2010) [82] and metastatic diseases (Vilhauer, 2010) [79]. It may also have been a problem in the Nicholas 2007 [81] intervention for parents of technology assisted children with lung disease who were assigned to dyads to exchange support. Additionally, several of the reviews (Hoey 2008, Campbell et al., 2004) [47, 83] suggested that unmoderated PS for those with high stress may be unproductive. Earlier findings of Helgeson [84] as well as two RCTs included in the review by Campbell [47] showed negative or mixed results of peer-led support groups. In contrast, however, PS interventions moderated by professionals have shown striking effects on quality of life and, in some cases, survival among women with metastatic disease (co-lead by psychiatrists or social workers with a counselor with breast cancer in remission) [85–87] and among newly diagnosed breast cancer patients (delivered by clinical psychologists) [88, 89]. It appears that key combinations of seriousness of problem, emphasis on emotions, peer-to-peer support, and roles of professionals need to be elucidated as they may yield either positive or adverse effects.

Two studies with diabetes failed to show overall benefits of PS versus control conditions, but nevertheless showed important benefits among subsamples. In the Chan 2014 study [70], all participants received standardized clinical care that included initial assessments, reports on medication adherence, self-monitoring, weight, diet, physical activity for patients and treatment intensification recommendations for physicians, and periodic reports to patients providing status updates and recommendations [90]. The addition of PS did not improve clinical indicators. However approximately 20% of the sample were above norms for depression, anxiety, and stress and also accounted for highly disproportionate rates of hospitalization. In this 20%, peer support not only reduced distress substantially, but also lowered rates of hospitalization to the levels of individuals not similarly distressed. The second diabetes study showing effects of peer support in a subsample was that of Simmons 2015 [67]. In spite of modest participation rates, noted above, assignment to group PS (with or without individual PS) was associated with significantly (*p* = 0.008) greater reductions in systolic blood pressure than other conditions.

Summarizing, the null or negative findings identified appear to be attributable to methodological problems, identifiable problems in how PS was implemented or promoted to those intended, or populations for whom unmoderated PS may be inappropriate. Further, in spite of lack of overall differences between PS and controls, several studies document apparent important benefits within subsets of participants.

### Cost effectiveness

Although cost savings and related outcomes have not been widely evaluated in the studies reviewed, a number of papers in the field have documented cost-effectiveness of PS [15, 29, 69, 91–96]. The review of Hunt 2011 [97] identified cost savings through reductions in emergency care and reductions in hospitalizations and Medicaid costs among adults with diabetes.

The paper by Forchuk and colleagues (2005) [98] reported remarkable cost savings through an intervention to improve quality of life and post discharge status of individuals with chronic mental illness in Canada. In addition to one year of PS post discharge, the intervention included continuity of contact between in-patient and post-discharge community staff**.** “At a rate of $632.30 CDN [Canadian dollars] per day cost for a bed in a psychiatric hospital, the people in the intervention group consumed $12,212,242 CDN less in hospital services than the control group, prior to discharge.” (p. 562). However, the $4400 CDN per person reduction in hospital and emergency services was only of borderline significance (*p* = 0.09) as variance in hospital cost data compromised sensitivity of analyses. Nevertheless, the effect of the post-discharge program on pre-discharge costs was suggested by ward staff reports; “…with the support that the [program] provided they felt comfortable discharging clients earlier…” (p. 562). Clearly, cost effectiveness and related analyses are important areas for growth of research on PS.

One of the studies identified through the extended review of peer support in diabetes also led to a cost-effectiveness analysis [99]. It used the differences attributable to peer support in an initial study (Prezio, 2013, 2014) [63, 100] and the Archimedes Model to estimate incidence of diabetes complications over twenty years. This led to an estimated incremental cost in the PS condition of $355 per quality adjusted life year gained. For reference, $50,000 per quality adjusted life year is often viewed as a benchmark for good value [91].

### Reach of PS

As in the promotion of breastfeeding of Graffy 2004 [77] in which no further contact with new mothers who had left the hospital would occur unless they contacted the peer supporters, simply inviting individuals to contact peer supporters may be insufficient to bring about such contact. More proactive approaches to offering PS may be necessary, especially among underserved groups whose experiences with the health care system may leave them understandably suspicious of new interventions. For example, a combination of persistent but low-demand contact established working relationships with “Asthma Coaches” among 89.6% of low-income, predominantly African American single mothers of children who had been hospitalized for their asthma [13]. In Pakistan, the successful “Lady Health Worker” intervention for post-partum depression [101] mitigated the impact of factors such as lack of financial empowerment on depression which, in the absence of intervention, sharply differentiated those becoming depressed [102].

In this regard, it is perhaps of significance that, of the 12 studies identified in the present review that were conducted in World Bank designated low-income economies (Bangladesh – 4, Mozambique – 1, Uganda – 1), lower-middle-income economies (Pakistan – 4), and upper-middle-income economies (Brazil – 1, South Africa – 1), 10/12 (83.3%) were RCTs in contrast to 38/54 (70.4%) of those from high-income economies. Nevertheless, all 12 of those from the low-income, low-middle, and upper-middle-income economies showed significant within- or between-group differences favoring PS, in contrast to 42/54 (77.8%) of those from high-income economies. It appears that PS can be well implemented and effective in under-resourced settings.

### Peer support in diabetes management

In the extended review in diabetes, a similar proportion of studies using RCT or Other Controlled Designs and objective or standardized outcomes reported significant effects of peer support (20 of 24 or 83.3%) as in the initial, broader review (36 of 43 or 83.7%). It is noteworthy also that the average reduction of HbA1c observed over the 23 studies reporting these data was 0.76 points. This is well above a benchmark 0.5 points generally considered to be clinically meaningful.

Two recent reviews have also examined peer support in diabetes management. Palmas and colleagues [59] restricted their review to RCTs evaluating changes in HbA1c in comparisons of community health workers versus usual care over a period of at least 12 months. They identified nine studies meeting these criteria with a standardized mean difference in change in HbA1c for intervention versus usual care of 0.21 (95% confidence interval = 0.11–0.32). In another 2015 review, Zhang and colleagues [103] examined 20 RCTs and found a pooled effect on HbA1c of −0.16 points (95% confidence interval of −0.007 to −0.25 points, *p* < 0.001). This review examined differences according to several categories of peer support providers. The greatest difference between changes in intervention and control groups (−0.49 points, 95% confidence interval − 0.12 to −0.86 to) was for interventions “provided by patients themselves to help each other or to share experience together in a group, usually with no specific leader during the intervention,” however, there were only two studies in this category [56, 104]. Substantial effects (−0.35 points, 95% confidence interval = −0.16 to −0.54) were also found for interventions “provided by nonprofessionals like community health workers, medical assistants, or community lay workers who had similar background or shared similar local culture with patients.” Smaller effects (−0.08 points, 95% confidence interval = −0.03 to −0.18) were found for interventions “led by one or several peer coaches, peer leaders, peer educators, peer supporters or peer mentors who were usually also patients but had received relevant training.” It should be noted that the effects on HbA1c in these two meta-analyses reflect the difference in changes between peer support and control conditions, while the 0.76 point mean difference identified in the present review only reflects the pre-post change within the condition receiving PS.

### Limitations

Because of the aforementioned variety of a) designs, b) health problems addressed, c) pertinent outcomes, and d) intervention strategies, the present review has not graded studies according to general criteria of quality of research design or risk of bias beyond the categorizations of type of design (RCT, Other Controlled, Within-Group Pre-Post, and Other), and type of outcome (objective, standardized, nonstandardized). Also, it has not included meta-analytic characterization of results of studies reviewed. Continued review of research on PS will benefit from such approaches. These limits were accepted in the interest of capturing the variety of PS programs and outcomes around the world.

It is important to recall the restriction of the present review to interventions applying PS to complex health behaviors needing to be sustained over time and thus the exclusion of interventions focused on isolated or single behaviors, such as undergoing mammography or other screening procedures. This restriction was not based on any supposed lack of importance of these other applications of PS, but only on recognition that PS interventions and factors key to their success may be very different in the two arenas. Evidence indicates effects of PS in screening, [28, 105, 106] effects that complement those identified here.

The initial broad review of articles and reviews only through July 15, 2011 is a shortcoming of the paper. In addition to the extended review of papers in diabetes through 2015, subsequent papers and reviews have continued to document the effects of peer support in a manner similar to those noted here (e.g., [25, 29, 107]).

## Conclusion

Across 65 identified research studies, 24 reviews, and the 30 studies on peer support in diabetes, and including under-resourced countries and health care systems, PS is shown to have effects in encouraging and helping to sustain a variety of complex health behaviors in prevention and disease management and in areas such as cardiovascular disease, HIV/AIDS, diabetes and other chronic diseases, maternal and child health, and mental health. These findings add to growing evidence [25, 29, 107] that PS is an effective tool to improve health outcomes.

## Additional files

Additional file 1: Details of Studies from Systematic Review of Peer Support. Details of studies reviewed including peer support intervention, population and health problem to which applied, research design, characterization of strongest outcome measure, and outcomes observed. (DOCX 121 kb)

Additional file 2: Details of Studies Included in Review of Peer Support in Diabetes Management. Details of studies reviewed including population to which applied, whether intervention was for diabetes prevention or management, research design, characterization of strongest outcome measure and outcome observed, and pre and post Hemoglobin A1c values or changes in Hemoglobin A1c values. (DOCX 130 kb)

## Abbreviations

BMI: Body-mass index
HbA1c: Hemoglobin A1c
NS: Nonsignificant
PS: Peer support
RCT: Randomized controlled trial
SBG: Significant between group
SWG: Significant within group
