# Supplementary material for: Peer support of complex health behaviors in prevention and disease management with special reference to diabetes: systematic reviews
Source: Clin Diabetes Endocrinol. 2017 May 25;3:4. doi: 10.1186/s40842-017-0042-3 (PMC5471959; doi:10.1186/s40842-017-0042-3)
Supplement: Supplementary file 1 — Details of Studies from Systematic Review of Peer Support. Details of studies reviewed including peer support intervention, population and health problem to which applied, research design, characterization of strongest outcome measure, and outcomes observed. (DOCX 121 kb) [file 40842_2017_42_MOESM1_ESM.docx]

**Additional File 1: Details of Studies from Systematic Review of Peer Support**

**Abbreviations:**

CHW = community health worker

Cx = counseling

ED = emergency department

HbA1c = hemoglobin A1c measure of metabolic control in diabetes

PS = Peer Support Treatment or Intervention

UC = usual care

**Designs**

ATC = alternative treatment control

RCT = Randomized controlled trial

WGP-P = within group, pre-post design

**Outcomes**

NSt = nonstandardized measure of outcome

Ob = objective measure of outcome

SBG = significant, between-groups difference favoring PS

St = standardized measure of outcome

SWG = significant, within-groups difference for PS

**Health Problems, Prevention/Management**

BH – Mental health, including post-partum depression

CVD – Cardiovascular disease including Heart Failure and general prevention through any or all of diet, exercise, blood pressure control

DAC – Addiction, Drug, Alcohol, Cigarette Smoking

DM – Diabetes, including support for parents of youth with diabetes

HA – HIV/AIDS

HF = Heart Failure

MCH – Pregnancy, Childbirth, Pre- and Post-natal Care, including breast feeding

OCD – Other Chronic Disease, including Asthma (including support for parents of youth with asthma), Cancer (including survivorship), COPD

OHP - other health promotion (e.g., smoking cessation)

Prev = prevention; Mgmt - disease management

| **1s Author, Year** (Full references at bottom of document)  **Intervention Description** | **Health Problem/ Prev, Mgmt Setting or Population** | **Design** | **Reach and Sample Description** | **Strongest Outcome** |
| --- | --- | --- | --- | --- |
| Auslander, 2002  Group, individual nutrition counseling from peer educators | Diabetes/Prev/  African American women | RCT: PS vs control (workbook, half-day workshop) | Of 398 completing pre-test, 104 dropped out | SBG, St (dietary patterns, p<.0001; dietary fat, p<.001) |
| Babomoto, 2009  CHW intervention of educational sessions and follow-up phone calls | Diabetes/Mgmt/ Latino adults | RCT: PS vs nurse case management vs UC | Of 354 eligible, 318 enrolled in study | SBG, St (Self reports of diet, Rx adherence) |
| Balcazar, 2005  *Promotores* conduct educational sessions, home visits, telephone follow up | CVD/Prev  Latino adults | WGP-P | 223 families (320 individuals) participated (no discussion of # eligible) | SWG, St (CVD risk behaviors) |
| Baqui, 2008  Home visits from CHWs | Birth outcomes/ Prev | RCT: Cluster randomization to PS vs group meetings led by trained providers vs UC | Of 58,588 eligible, 1767 declined to participate and 10,377 did not participate for other reasons | SBG, Ob (Neonatal mortality reduced 34% in PS, 29.2 per 1000) |
| Bernstein, 2009  From ED, 20-30 min structured conversation w/ peer + booster phone call 10 days later | Marijuana use/ Prev | RCT: Written info and 12 mos assmt vs Written info and 3, 12 mos assmt vs PS | Of 352 eligibles, 52 refused and 63 missing/no opportunity to complete consent. | SBG, St (use in previous 30 days, p = 0.053) |
| Bhutta, 2008  LHWs and traditional birth attendants providing basic newborn care | Birth outcomes/ Prev | Quasi experimental, assigned by village | Offered as part of routine care in PS villages | SWG, NSt (clinical records of stillbirths, neonatal mortality) |
| Bhutta, 2011  LHWs provided group educ and indiv support; traditional birth attendants provided basic newborn care | Birth outcomes/ Prev | Cluster randomized with 16 clusters randomized to enhanced LHW or usual care | Offered as routine care in intervention villages. Conducted 63% of planned group sessions with 24% of mothers | SBG, Ob (stillbirths from registry reduced to 39.1 per 1000 births vs 48.7, *p* = 0.006; neonatal mortality = 43.0 per 1000 vs 49.1, *p* = 0.02) |
| Broadhead, 2002  HIV+ “advocates” provided support to each other | HIV+/Mgmt/  HIV+ drug users | Pilot study with 15 participants | Of 15, 14 engaged in reciprocal support, kept 80% of apptmts with each other | SWG, NSt (kept 84% of healthcare apptmts, reduced needle sharing) |
| Chang, 2010  PS for patients on antiretroviral Tx (ART) through counseling in clinics and biweekly home visits | HIV/AIDS/ Mgmt/ AIDS clinics in Uganda | RCT: Cluster randomized 15 AIDS clinics 2:1 to PS vs UC | All ART patients eligible. PS reached 96% ≥ 1X, averaged 1.1 /pt/month | SBG, Ob (Sig decline in virologic failure relative to controls at 96, 144, 168, and 192 wks, *p* = 0.016) |
| Chapman, 2004  Peer counseling: pre- and postnatal visits and telephone contact as needed | Breast feeding/ OHP/Low-income Latina women | RCT: routine breastfeeding alone or with peer counseling | Of 424 assessed for eligibility, 205 were ineligible, 45 refused, and 219 were randomized | SBG, NSt (initiating and sustaining breastfeeding) |
| Chen, 2010  PS for chronic mgmt. of diabetes, hypertension in clinical setting | Diabetes, Hyptertension/ Mgmt/ Pts of safety net provider | PS worked with patients of 1^st^ year residents, Compared to pts of 2^nd^, 3^rd^-year residents, and to present and prior year by EMR and chart audit | Of 146 patients eligible, 7 refused, 27 moved | NS, NSt (greater impvmt in BP, HbA1c, LDL in PS than control, but NS) |
| Clark, 2012  PS by former pts to promote PA among pts in cardiac rehab | CVD/ Mgmt | WGP-P | Of 109 eligibles, 79 joined (older, more women compared to non-joiners) | SWG, St (Self-reported physical activity) |
| Dale, 2009  Telecare motivational support for diabetes mgmt provided by PS | Diabetes/Mgmt/ Pts of cooperating practices in England | RCT: Telecare support by specialist nurses vs by PS vs UC | Of 447 invited to participate, 231 consented | SWG, Ob (HbA1c from 8.4% to 8.0% in PS group) |
| Darmstadt, 2010  2 antenatal visits and 4 postnatal visits from community health workers for mothers and their newborns | MCH/Prev/  in Mirzapur, Bangladesh | RCT: cluster RCT compared intervention to mothers receiving standard | Across several survey waves %s with 2 prenatal visits ranged from 83.8% to 91.0%; % with at least one post-natal visit, from 75.5% to 83.7%. | SBG, NSt Significantly greater increases on measures of knowledge of maternal and neonatal danger signs and several antenatal and postnatal practices |
| Dennis, 2002a, 2002b  Telephone based mother-to-mother peer support for breastfeeding | Breastfeeding/ Prev /Primiparous women | RCT: PS vs UC | Of 359 eligible, 101 declined, most because of sufficient existing support | SBG, St (continued and exclusive breastfeeding) |
| Dennis, 2003  Pilot of mother-to-mother telephone peer support for postpartum depression tested in Dennis, 2009, below | Postpartum depression/Prev | RCT: PS vs UC |  | SBG, St (Edinburgh Postnatal Depression Scale, p = 0.02) |
| Dennis, 2009  Telephone based mother-to-mother peer support for postpartum depression | Postpartum depression/Prev | RCT: PS vs UC | Of 1740 eligible, 701 enrolled. Nonparticipants either refused, agreed but could not be contacted, or did not meet additional eligibility criteria. | SBG, St (14% vs 25% point prevalence of depression at 12 weeks post-partum) |
| Di Meglio, 2010  7 telephone support calls from trained teenage mothers in Rochester, New York | Breastfeeding/ Prev | RCT: Breastfeeding mothers randomly assigned to intervention or standard care over 5 weeks post-birth. | 90 eligibles identified through Maternity Wards. 78 agreed to participate. 19/38 completed intervention. 24/40 controls completed assessments | SBG, St Mothers in intervention more likely to report exclusive breast feeding (not using supplements) than control mothers (p=.0004) |
| Dorgo, 2009  Structured exercise training administered by peer supporters | Physical activity/ Prev  Older adults | RCT: PS vs exercise training by kinesiology students | Enrolled 149 | SWG, St (physical, mental, social functioning on SF 36) |
| Elder, 2005, 2006, 2009  Tailored print materials and personalized dietary counseling from *promotoras* | Healthy diet/Prev/ Latina women | RCT: PS+tailored print, tailored print materials alone, non-tailored print materials | 357 women recruited and completed baseline home visits | SBG, St (diet and behavioral strategies for healthy diet) |
| Fisher, 2009  Asthma Coach for mothers of children hospitalized for asthma | Asthma/Mgmt/ Urban children covered by Medicaid | RCT: PS vs UC | Of 306 eligible, 106 unreached (no-answer, disconnected), 9 refusal, 191 randomized | SBG, Ob (relative risk of rehospitalization = 0.61 relative to UC) |
| Flowers, 2002  Gay Men’s Task Force (bar-based, peer-led educational interactions, referrals, free phone hotline) | HIV/AIDS/Prev | Comparison Groups: PS in Glasgow vs UC in Edinburgh  Those reporting PS contact versus others | In Glasgow, 424 of 1380 completing surveys reported direct contact with PS | SWG, NSt (among those who reported contact with PS, improvement relative to whole group at baseline in Hep B vaccination, HIV testing |
| Forchuk, 2005  Continuity of contact with in-patient staff plus PS | Chronic mental illness/Mgmt | RCT: Cluster assignment to PS + Continuity of Contact vs UC | 390 patients recruited at time of discharge | SBG, St (quality of life) |
| Fox, 2007  Continuous Quality Improvement with CHW education in homes and the community | Asthma/Mgmt/ Children | WGP-P in seven study sites | 541 enrolled in longitudinal cohort | SWG, NSt (reduction in hospitalizations – 8.6% to 1.7% of cohort; emergency care – 26.9% to 8.6%; frequent use of rescue meds – 42.3% to 11.8%) |
| Graffy, 2004  Postnatal in-person visits plus phone calls on request | Breast feeding/ Prev/Women registered for maternity care | RCT: PS vs UC | Of 5193 registered for maternity care, 4473 excluded (not pregnant, moved away, not seen, registered late, questionnaires unreturned, not recruited, ineligible), 720 randomized. | Nonsig, St: No differences in breast feeding between groups up to 4 mos post-partum |
| Greenhalgh, 2011  Story sharing groups, 2 hours biweekly for 6 months | Diab/ Mgmt  Socioeconomically disadvantaged area of London | RCT: PS vs UC | 157 agreed to participate of 285 who met criteria | SBG, St (differences on Patient Enablement, p < 0.005) |
| Haider, 2000  Peer counseling from mothers w/ breastfeeding experience in ante- and post-natal period | Breast Feeding/ Prev  Women in Bangladesh | RCT: PS vs UC | Of 783 eligible, 726 randomized (92.7%)  In PS, 323 of 337 eligibles enrolled (95.8%) | SBG NSt (84% vs 30% exclusive breastfeeding at day 4 post-partum, 70% vs 6% at 5 mos) |
| Heisler, 2010  Reciprocal peer support plus nurse care management | DM / Mgmt Male US armed services veterans with diabetes | RCT: randomized to 3-hr session to set diabetes-related goals, receive peer communication skills, and be paired with age-matched peers vs enhanced usual care of 1:1 consultation with care manager, self-care materials, optional phone or face-to-face follow-up. | 927 of 1699 (54.6%) declined participation. Randomly assigned 244 males to intervention (n=126) or control (n=119). 90% of peer pairs had at least one conversation | SBG Obj Intervention had lower HbA1c at 6 mos (P=.004) compared to control. More intervention patients started insulin (P=.02). |
| Hoybye, 2010  Week long rehabiliation plus lecture on use of the internet for support and access to an Internet support group. | OCD Cancer / Mgmt with cancer survivors | RCT Randomized by week to intervention or only week-long rehabilitation program | 58 groups of cancer survivors (n=921). 438 in control and 366 in intervention completed baseline interviews (n=804, 87.3%) . 633 (68.7%) completed program and follow up. | SWG St increase in vigor for intervention group |
| Hunkeler, 2000  Nurse telehealth including medical advice and emotional support + PS in-person and by telephone | Depression/Mgmt | RCT: PS + Nurse telehealth vs Nurse telehealth alone vs UC | Of 370 eligible, 302 enrolled (81.6%) Of 62 in PS, 11 refused, 9 never had peer contact. Of remaining 42, 11 had 1 contact, and only 6 had at least 1 face-to-face | NS St - nonsignificant between group differences; actual values not reported |
| Jason, 2010  4-month buddy intervention | Chronic Disease /Mgmt | RCT: 4-month buddy vs usual care | 30 recruited by referral, from support groups, and from newsletter | SBG, St – fatigue and vitality on MOS SF-36 |
| Kaplan, 2011  Internet PS by listserv or by bulletin board | Mental Health/Mgmt Adults with schizophrenic spectrum or affective disorders | RCT: Internet PS by listserv, Internet PS by bulletin board, Waitlist control | From website and e-newslist, 2,057 expressed interest, 952 provided information, 336 eligibile, & 300 gave consent and were randomized | NS, St  Modest participation: "Approximately 1/3 … in … bulletin board condition created an account but never logged in." |
| Krieger, 2002, 2005  PS provided in-home assessment, education, support, resources | Asthma/Mgmt/ Children | RCT: high and low intensity PS | Of 1116 potentially eligible from med records, 714 reached and 274 randomized. 267 not eligible, 90 refused screening, 68 refused/cancelled baseline. | SBG, St. for greater decreases in intense PS for self-reported urgent care in previous 2 months (p = 0.026) and symptom-free days (p = 0.138) |
| Krieger, 2009  PS for home environmental assessments, asthma ed., social support, and asthma control resources | Asthma/Mgmt/ Children | RCT: Nurse led asthma educ plus referrals to community resources alone and with PS for home assessments, asthma education, social support, and asthma-control resources. | Of 1474 with Dx of asthma, 969 caregivers reached, and 309 randomized. 283 did not meet criteria, 252 refused, 125 did not complete enrollment | SBG St for caregiver’s QOL (p = 0.05)  SBG NSt for Sx-free days (p = 0.05) |
| Landers & Zhou, 2011  Community mental health and peer support | MH/Mgmt  Medicaid consumers identified with both community mental health and peer support claims | Control Non-RCT: Comparison group created on 2:1 ratio with patients with community mental health but no peer support claim, matched on gender, race, age group, urban/rural residence, and principle diagnosis |  | SBG Obj Those with peer support claim had greater likelihood of crisis stabilization (OR = 1.345, p=<.05), lower but not statistically significantly odds of hospitalization (odds = 0.871). Among those without stabilization, peer support linked to reduced odds of hospitalization (0.766, p=<.01). |
| le Roux, 2010  Mentor Mothers visiting mother-child dyads for 12 months supporting problem solving around nutrition | MCH / Mgmt/Prev among 500 underweight children, aged 0-5 in South African neighborhoods | RCT compared weight gain among children exposed to intervention versus controls receiving weight measurement only. | 788 mother-child dyads assigned in 2:1 random sequence to intervention (n=536) or control (n=252). 90% of dyads received at least one follow-up visit. 48 (4.0%) children were not weighed or passed away in intervention, 55 (21.8%) in control. | SBG Obj 43% of intervention children were rehabilitated to acceptable weight over 12 months as compared to 31% of controls (P<.01). |
| Leite, 2005  PS Home visits to promote breastfeeding | Breast feeding/OHP/ Mothers of low birthweight babies | RCT: PS vs UC | Of 3500 eligibles, 1003 agreed and were randomized | SBG St for exclusive breast feeding 4 months post-partum (24.7% vs 19.4%; p = 0.04); partially breastfed (37.2%, 30.2%; p = 0.02); artificially fed (20.1%, 33.4%; p < 0.001) |
| Levine, 2003  Nurse-supervised PS provided monitoring, educ, counseling, soc sup and comm outreach for BP mgmt. | Hypertension/ Mgmt/urban African Americans | RCT: High intensity PS (more than 5 visits) vs low intensity PS (one visit) | Of 2736, 2196 consented, of whom 817 were hypertensive of which 789 agreed to participate | SWG Ob Over 26 months, BP fell 148 to 138 mm (p < 0.05) and 89 to 82 mm (p < 0.05) in High Intensity, but returned to baseline by 40 months |
| Mannan, 2008  PS for care practices among new mothers, including breastfeeding | Breastfeeding/ OHP/ rural Bangladesh | Natural experiment to assess value of visits 1-3 days post partum: Among those receiving PS, compared those with 1st visit within 1-3 days versus 6-7 days post partum | 13,912 births within arm of cluster randomized study examining PS. After exclusions for stillbirths, abortions, deliveries outside area, and schedules of PS visits not allowing assessment of visit 1-3 days post partum, 3,495 included | SBG St: Overall feeding difficulties – 6% vs 34%, p < 0.001 on standardized algorithm for assessment adopted by project; lower scores on each of 6 factors of measure of feeding difficulties, p < 0.001. |
| May, 2006  “Buddy” support among participants in group smoking cessation program | Smoking cessation/ OHP  DAC, M | Group randomization to smoking cessation group intervention with/without buddy support starting at quit date | Of 630 participants in 34 groups, 96 excluded because of nonattendance at 2^nd^ meeting (quit day) when buddy introduced | Nonsig Ob measure smoking status.  Borderline betw grps at 1 week post-quit (*p* = 0.06) |
| McInnes, 2000  PS and awareness activities for breastfeeding | Breastfeeding/ OHP  MCH, Prev | Quasi experimental comparison of 2 clinics assigned to PS vs UC | Of 995 across both clinics, 55 lost or withdrew prior to baseline assessment | SBG NSt self report measure of initiating breastfeeding |
| Muirhead, 2006  PS added to midwife support, group support, workshops for breastfeeding | Breastfeeding/ OHP  MCH, Prev | RCT of midwife support for 10 days, groups, workshops to encourage breastfeeding with/without PS | 225 of 284 approached agreed to randomization | Nonsig NSt measure of breastfeeding |
| Nelson, 2011  Asthma Coach for 18 months for parents of children with asthma | MCH OCD / Mgmt  Children with asthma with acute emergency visits | RCT ofCoach vs usual care | 122 Coach parents and 125 control parents enrolled. Estimated 10% of parents approached declined | SBG NSt From records audits, Asthma monitoring visits in 2 yrs after enrollment more likely in Coach than usual care |
| Nicholas, 2007  PS for parents of children with chronic lung disease requiring technological assistance | Chronic lung diseases/ Mgmt/ Parents of children requiring care  OCD, M (COPD) | Qualitative and descriptive study of cases drawn from patient database | 34 parents of children in database | Nonsig St measures of coping with illness Qualitative findings identify benefits of intervention |
| Olson, 2010  PS for breastfeeding | Breastfeeding/  OHP  MCH, Prev | 336 who signed up for service and were contacted by PS compared to 654 not contacted | Pregnant women recruited through WIC program in 5 Michigan counties.  Numbers offered opportunity to sign up not reported | SBG, NSt measures of breast feeding |
| Omer, 2008.  PS enhanced by risk communication tools for pre- and postnatal care | Prenatal and postnatal care/ OHP/ Pakistan  MCH, Prev | Cluser randomization of communities to enhanced PS with risk communication tools vs standard PS | PS as part of Lady Health Worker services in communities  Those in communities with enhanced PS more likely to receive PS | SBG NSt interview measures of use of colostrum and breastfeeding. |
| Parent, 2000  PS modeled successful recovery and addressed pt’s questions 1 day pre- and 5 days and 4 weeks post CABG surgery | CVD (CABG recovery)/ Mgmt/ Canada | Patients randomized to information on surgery, recovery vs information plus PS | 67 of 70 agreed to participate 56 of 67 completed | SBG St on anxiety, self-efficacy, and general activity Possible bias by survey admin 1 hour after PS contacts and by study coordinator |
| Patterson, 2010  Peer support emphasizing personal achievement, planning leisure activities, community-based participation, adjustment to daily life facilitated by key group members | OCD (stroke)/ Mgmt  Stroke survivors | RCT 21 in peer support only group and 22 in weekly 60-minute exercise group + peer support |  | SWG St Both groups improved on standardized measure of time devoted to daily tasks at home, Home Functioning Questionnaire |
| Pearson, 2007  Directly observed treatment (DOT) for HAART delivered by PS 5 days per week for 6 weeks | HIV/AIDS/ Mgmt/ Mozambique | RCT to comprehensive adherence support that also includes PS vs comprehensive adherence support plus PS delivered DOT | Of 683 starting HAART, 25 not referred, 225 not eligible, 83 refused. 350 randomized of whom 1 refused DOT | SBG St “commonly used question” re: number doses missed in previous 7 days at 6 and 12 mos |
| Preyde, 2003  Mother-mother support, group meetings for mothers of preterm infants in neonatal ICU | MCH (Maternal distress)/ Mgmt/ Mothers of preterm infants | Standard medical and social work services in comparison hospital vs Standard plus mother-mother, group support in intervention hospital | Of 41 eligibles, 7 declined, child of one died, 1 lost to follow up | SBG St on Parental Stressor Scale at 4 wks and State Anxiety and Beck Depression Inventory at 16 weeks post-partum |
| Primomo, 2006  PS: education, action plans, home assessments and telephone follow up | OCD (Asthma)/ Mgmt/ Families of children with asthma | Pre-post program evaluation | Of 197 families receiving services, 105 consented and 60 completed follow-up survey | SWG St on caregiver quality of life as well as use of action plan, and caregiver-reported hospitalizations |
| Rahman, 2008  “Thinking Healthy” adaptation of problem solving and cognitive behavior therapy for PS | BH (Post-partum depression)/ Mgmt/ New mothers in Pakistan | Cluster randomized design Mothers identified as depressed in 3^rd^ trimester received PS or usual care from Lady Health Workers | 1967 in 3^rd^ trimester in PS communities; 1787 completed baseline; 463 (25.9%) met criteria for depression; 418 (90.3%) and 412 (89%) assessed at 6, 12 mos post partum | SBG St 23% vs 53% depressed at 6 mos post-partum by psychiatric assmt (DSM-IV); 27% vs 59% at 12 mos, *p*s < .0001 |
| Rhodes, 2009  Within soccer teams, *Navigantes* promoted condom use | HIV+/ Prev/ Latino men in No Carolina | 15 Intervention teams and 15 Non-randomized comparison teams | From 89 teams, 15 intervention and 15 comp selected. 222 men randomly selected for post-test | SBG NSt 65% vs 41.3% consistent condom use in prev 30 days, *p* <.01. Also HIV testing (64.4% vs 41.8%), knowl of HIV transmission/prev (74.1% vs 43.5%) and self efficacy for condom use (55.6% vs 38.2%) |
| Riegel, 2004  Home visits, telephone calls, joint outings, modeling, support groups vs UC support groups | CVD (Heart Failure)/ Mgmt/ Elderly males | RCT of patients Dx with Heart Failure, cognitively intact, and to be discharged to home | 63% of eligible refused. Reasons included: “not interested” (49.7% of refusers), “too busy” (14%), desire to be left alone (7.4%), and “I’ve done OK so far” (6.7%) | SBG St on total scores and subscores for confidence and management (Self-Care of Heart Failure Index), *p*s < 0.02 to 0.04. |
| Rowe, 2007  PS + community-oriented interv + jail diversion to reduce alcohol/drug use, criminal justice charges | DAC (Alcohol)/ Mgmt/ Those facing criminal justice charges | RCT PS + community-oriented interv + jail diversion vs. standard clinical treatment + jail diversion services | 114 participants. #s eligible, refused, etc. not included | SBG St (alcohol use, *p* < 0.05)  SWG Obj (criminal justice charges, *p* < 0.05) |
| Sacco, 2009  Telephone PS for diabetes mgmt | DM/ Mgmt | RCT: PS vs UC | Of 98 eligibles, 9 not reached and 10 declined randomization. 62 of 79 returned pre-tests, were randomized | SBG St (PHQ-9 depression, *p* < .05; Diabetes Sx Checklist, *p* < .01; Diabetes Self-Care Activities, *p* < .001) |
| Salzer, 2010  Unmoderated peer support listserv | OCD (BrCA)/ Mgmt | RCT: PS vs internet-based educational control | Of 408 responding to initial calls, 222 did not provide additional info. Of 79 eligibles, 1 withdrew | Counter St Controls exceeded PS on QOL (FACT-B at 4, 12 mos, *p* < .05) |
| Simmons, 2008  PS plus social mobilization, subsidized GP visits, referral to exercise coach vs UC | DM/ Prev/ Maoris in New Zealand | Pilot of larger study. Pre-Post in PS vs Pre in comparison group | 160 from selected districts of 5240 recruited for larger, cluster-randomized design | SWG Obj weight loss among those with impaired glucose tolerance, impaired fasting glucose (*p* < .01) |
| Simoni, 2007  PS for adherence via biweekly group meetings and weekly indiv contact | HIV/AIDS/ Mgmt | RCT: PS vs UC | 136 randomized to PS vs UC 53% of eligibles declined: lacking interest, too busy, transportation or “asocial” | NS NSt (viral load from medical record abstractions, electronic adherence monitoring) |
| Smith, 2011  9 PS group sessions over 2 years | DM/ Mgmt/ Patients of General Practices in Ireland | Cluster RCT by practice: Standardized diabetes care vs Standardized + PS | In PS practices, 30% (89/292 eligibles) declined (not interested, etc.)  18% attended 0 grp meetings; mean = 5/9 | NS Obj (HbA1c, BP, chol, BMI) |
| Staten, 2004  Biweekly-monthly phone calls and monthly walks organized by CHWs | OCD (general chronic disease)/ Prev/ uninsured Hispanic women aged ≥ 50 years | RCT: Provider Cx (PC), PC + health ed (HE), PC + HE + PS. Rectmt. From Nat Br Cerv Ca Early Det Prog | 14% (68/478) of eligibles declined 66.7% (217/326) completed pre- and 12-mos post assessment | SWG Ob (SBP, total chol, % high BP) and St (PA and F&V) |
| Sullivan, 2004  Support by peer mothers for mothers of children newly Dx’d w/ type 1 DM | DM/ Mgmt mothers and children with type 1 DM | Intervention vs Wait-list control receiving trtmt after 6 mos | Of 54 approached, 49 mothers agreed to participate, 42 were reached and randomized. | SBG St (diabetes-related concerns, *p* = 0.02, and impact on family *p* = 0.05) |
| Travis, 2010  Mutual telephone support via dyads with depression | BH (Dep)/ Mgmt/ Pts from VA and Comm Mental Hlth Ctrs | Within-group pre-post | 54 enrolled and 32 (59%) completed 12-week program | SWG St depression (BDI; *p* = .02), functional disability (*p* =.02), psychological health (SF-12; p<.001) QOL (QOL Questionnaire Short Form; *p* = .04). |
| Vilhauer, 2010  Unmoderated mutual web-based support | OCD (Breast CA)/ Mgmt | Randomized to Intervention or Wait-List Control | From treatment centers and > 900 mailings to patients, 42 replied and 31 eligible and randomized | SBG St Counter Wait-list improved more than PS on Eastern Coop Onc Grp Perf Status Scale Other measures: acceptance of and satisfaction with web-based support |
| Weber, 2007  Dyadic support to enhance self-efficacy, decrease depression | OCD (Prostate CA)/ Mgmt | RCT: PS vs UC | Of 152 eligible, 50 failed to respond/non interested, 21 out of catchment area | SBG St (depression, Geriatric Depression Scale, *p* =.005; self-efficacy Stanford Inventory of Cancer Patient Adjustment, *p* =.02) |

**References**

Auslander, W., Haire-Joshu, D., Houston, C., Rhee, C. W., & Williams, J. H. (2002). A controlled evaluation of staging dietary patterns to reduce the risk of diabetes in African-American women. *Diabetes Care, 25*(5), 809-814.

Babamoto, K. S., Sey, K. A., Camilleri, A. J., Karlan, V. J., Catalasan, J., & Morisky, D. E. (2009). Improving diabetes care and health measures among hispanics using community health workers: results from a randomized controlled trial. *Health education & behavior : the official publication of the Society for Public Health Education, 36*(1), 113-126.

Balcazar, H., Alvarado, M., Hollen, M. L., Gonzalez-Cruz, Y., & Pedregon, V. (2005). Evaluation of Salud Para Su Corazon (Health for your Heart) -- National Council of La Raza Promotora Outreach Program. *Preventing chronic disease, 2*(3), A09.

Baqui, A. H., El-Arifeen, S., Darmstadt, G. L., Ahmed, S., Williams, E. K., Seraji, H. R., et al. (2008). Effect of community-based newborn-care intervention package implemented through two service-delivery strategies in Sylhet district, Bangladesh: a cluster-randomised controlled trial. *Lancet, 371*(9628), 1936-1944.

Bernstein, E., Edwards, E., Dorfman, D., Heeren, T., Bliss, C., & Bernstein, J. (2009). Screening and brief intervention to reduce marijuana use among youth and young adults in a pediatric emergency department. *Acad Emerg Med, 16*(11), 1174-1185.

Bhutta, Z. A., Memon, Z. A., Soofi, S., Salat, M. S., Cousens, S., & Martines, J. (2008). Implementing community-based perinatal care: results from a pilot study in rural Pakistan. *Bulletin of the World Health Organization, 86*(6), 452-459.

Bhutta, Z. A., Soofi, S., Cousens, S., Mohammad, S., Memon, Z. A., Ali, I., et al. (2011). Improvement of perinatal and newborn care in rural Pakistan through community-based strategies: a cluster-randomised effectiveness trial. *Lancet, 377*(9763), 403-412.

Broadhead, R. S., Heckathorn, D. D., Altice, F. L., van Hulst, Y., Carbone, M., Friedland, G. H., et al. (2002). Increasing drug users' adherence to HIV treatment: results of a peer-driven intervention feasibility study. *Social science & medicine, 55*(2), 235-246.

Chang, L. W., Kagaayi, J., Nakigozi, G., Ssempijja, V., Packer, A. H., Serwadda, D., et al. (2010). Effect of peer health workers on AIDS care in Rakai, Uganda: a cluster-randomized trial. *PLoS One, 5*(6), e10923.

Chapman, D. J., Damio, G., Young, S., & Perez-Escamilla, R. (2004). Effectiveness of breastfeeding peer counseling in a low-income, predominantly Latina population: a randomized controlled trial. *Arch Pediatr Adolesc Med, 158*(9), 897-902.

Chen, E. H., Thom, D. H., Hessler, D. M., Phengrasamy, L., Hammer, H., Saba, G., et al. (2010). Using the Teamlet Model to improve chronic care in an academic primary care practice. *Journal of general internal medicine, 25 Suppl 4*, S610-614.

Clark, A. M., Munday, C., McLaughlin, D., Catto, S., McLaren, A., & Macintyre, P. D. (2012). Peer support to promote physical activity after completion of centre-based cardiac rehabilitation: evaluation of access and effects. *European journal of cardiovascular nursing : journal of the Working Group on Cardiovascular Nursing of the European Society of Cardiology, 11*(4), 388-395.

Dale, J., Caramlau, I., Sturt, J., Friede, T., & Walker, R. (2009a). Telephone peer-delivered intervention for diabetes motivation and support: the telecare exploratory RCT. *Patient Educ Couns, 75*(1), 91-98.

Dale, J., Caramlau, I., Sturt, J., Friede, T., & Walker, R. (2009b). Telephone peer-delivered intervention for diabetes motivation and support: the telecare exploratory RCT. *Patient education and counseling, 75*(1), 91-98.

Darmstadt, G. L., Choi, Y., Arifeen, S. E., Bari, S., Rahman, S. M., Mannan, I., et al. (2010). Evaluation of a cluster-randomized controlled trial of a package of community-based maternal and newborn interventions in Mirzapur, Bangladesh. *PLoS One, 5*(3), e9696.

Dennis, C. L. (2002). Breastfeeding peer support: maternal and volunteer perceptions from a randomized controlled trial. *Birth, 29*(3), 169-176.

Dennis, C. L., Hodnett, E., Gallop, R., & Chalmers, B. (2002). The effect of peer support on breast-feeding duration among primiparous women: a randomized controlled trial. *CMAJ : Canadian Medical Association journal = journal de l'Association medicale canadienne, 166*(1), 21-28.

Dennis, C. L., Hodnett, E., Kenton, L., Weston, J., Zupancic, J., Stewart, D. E., et al. (2009). Effect of peer support on prevention of postnatal depression among high risk women: multisite randomised controlled trial. *BMJ, 338*, a3064.

Di Meglio, G., McDermott, M. P., & Klein, J. D. (2010). A randomized controlled trial of telephone peer support's influence on breastfeeding duration in adolescent mothers. *Breastfeeding Medicine, 5*(1), 41-47.

Dorgo, S., Robinson, K. M., & Bader, J. (2009). The effectiveness of a peer-mentored older adult fitness program on perceived physical, mental, and social function. *J Am Acad Nurse Pract, 21*(2), 116-122.

Elder, J. P., Ayala, G. X., Campbell, N. R., Slymen, D., Lopez-Madurga, E. T., Engelberg, M., et al. (2005). Interpersonal and print nutrition communication for a Spanish-dominant Latino population: Secretos de la Buena Vida. *Health psychology : official journal of the Division of Health Psychology, American Psychological Association, 24*(1), 49-57.

Elder, J. P., Ayala, G. X., Slymen, D. J., Arredondo, E. M., & Campbell, N. R. (2009). Evaluating psychosocial and behavioral mechanisms of change in a tailored communication intervention. *Health Educ Behav, 36*(2), 366-380.

Fisher, E. B., Strunk, R. C., Highstein, G. R., Kelley-Sykes, R., Tarr, K. L., Trinkaus, K., et al. (2009). A randomized controlled evaluation of the effect of community health workers on hospitalization for asthma: the asthma coach. *Arch Pediatr Adolesc Med, 163*(3), 225-232.

Flowers, P., Hart, G. J., Williamson, L. M., Frankis, J. S., & Der, G. J. (2002). Does bar-based, peer-led sexual health promotion have a community-level effect amongst gay men in Scotland? *International journal of STD & AIDS, 13*(2), 102-108.

Forchuk, C., Martin, M. L., Chan, Y. L., & Jensen, E. (2005). Therapeutic relationships: from psychiatric hospital to community. *Journal of psychiatric and mental health nursing, 12*(5), 556-564.

Fox, P., Porter, P. G., Lob, S. H., Boer, J. H., Rocha, D. A., & Adelson, J. W. (2007). Improving asthma-related health outcomes among low-income, multiethnic, school-aged children: results of a demonstration project that combined continuous quality improvement and community health worker strategies. *Pediatrics, 120*(4), e902-911.

Graffy, J., Taylor, J., Williams, A., & Eldridge, S. (2004). Randomised controlled trial of support from volunteer counsellors for mothers considering breast feeding. *BMJ, 328*(7430), 26.

Greenhalgh, T., Campbell-Richards, D., Vijayaraghavan, S., Collard, A., Malik, F., Griffin, M., et al. (2011). New models of self-management education for minority ethnic groups: pilot randomized trial of a story-sharing intervention. *Journal of health services research & policy, 16*(1), 28-36.

Haider, R., Ashworth, A., Kabir, I., & Huttly, S. R. (2000). Effect of community-based peer counsellors on exclusive breastfeeding practices in Dhaka, Bangladesh: a randomised controlled trial [see commments]. *Lancet, 356*(9242), 1643-1647.

Heisler, M., Vijan, S., Makki, F., & Piette, J. D. (2010). Diabetes control with reciprocal peer support versus nurse care management: a randomized trial. *Annals of internal medicine, 153*(8), 507-515.

Hoybye, M. T., Dalton, S. O., Deltour, I., Bidstrup, P. E., Frederiksen, K., & Johansen, C. (2010). Effect of Internet peer-support groups on psychosocial adjustment to cancer: a randomised study. *Br J Cancer, 102*(9), 1348-1354.

Hunkeler, E. M., Meresman, J. F., Hargreaves, W. A., Fireman, B., Berman, W. H., Kirsch, A. J., et al. (2000). Efficacy of nurse telehealth care and peer support in augmenting treatment of depression in primary care. *Archives of family medicine, 9*(8), 700-708.

Jason, L. A., Roesner, N., Porter, N., Parenti, B., Mortensen, J., & Till, L. (2010). Provision of social support to individuals with chronic fatigue syndrome. *Journal of clinical psychology, 66*(3), 249-258.

Kaplan, K., Salzer, M. S., Solomon, P., Brusilovskiy, E., & Cousounis, P. (2011). Internet peer support for individuals with psychiatric disabilities: A randomized controlled trial. *Soc Sci Med, 72*(1), 54-62.

Krieger, J., Takaro, T. K., Song, L., Beaudet, N., & Edwards, K. (2009). A randomized controlled trial of asthma self-management support comparing clinic-based nurses and in-home community health workers: the Seattle-King County Healthy Homes II Project. *Archives of pediatrics & adolescent medicine, 163*(2), 141-149.

Krieger, J. W., Takaro, T. K., Song, L., & Weaver, M. (2005). The Seattle-King County Healthy Homes Project: A Randomized, Controlled Trial of a Community Health Worker Intervention to Decrease Exposure to Indoor Asthma Triggers. *American Journal of Public Health, 95*, 652-659.

Landers, G. M., & Zhou, M. (2011). An analysis of relationships among peer support, psychiatric hospitalization, and crisis stabilization. *Community mental health journal, 47*(1), 106-112.

le Roux, I. M., le Roux, K., Comulada, W. S., Greco, E. M., Desmond, K. A., Mbewu, N., et al. (2010). Home visits by neighborhood Mentor Mothers provide timely recovery from childhood malnutrition in South Africa: results from a randomized controlled trial. *Nutr J, 9*, 56.

Leite, A. J., Puccini, R. F., Atalah, A. N., Alves Da Cunha, A. L., & Machado, M. T. (2005). Effectiveness of home-based peer counselling to promote breastfeeding in the northeast of Brazil: a randomized clinical trial. *Acta Paediatr, 94*(6), 741-746.

Levine, D. M., Bone, L. R., Hill, M. N., Stallings, R., Gelber, A. C., Barker, A., et al. (2003). The effectiveness of a community/academic health center partnership in decreasing the level of blood pressure in an urban African-American population. *Ethn Dis, 13*(3), 354-361.

Mannan, I., Rahman, S. M., Sania, A., Seraji, H. R., Arifeen, S. E., Winch, P. J., et al. (2008). Can early postpartum home visits by trained community health workers improve breastfeeding of newborns? *Journal of perinatology : official journal of the California Perinatal Association, 28*(9), 632-640.

May, S., West, R., Hajek, P., McEwen, A., & McRobbie, H. (2006). Randomized controlled trial of a social support ('buddy') intervention for smoking cessation. *Patient Educ Couns, 64*(1-3), 235-241.

McInnes, R. J., Love, J. G., & Stone, D. H. (2000). Evaluation of a community-based intervention to increase breastfeeding prevalence. *J Public Health Med, 22*(2), 138-145.

Muirhead, P. E., Butcher, G., Rankin, J., & Munley, A. (2006). The effect of a programme of organised and supervised peer support on the initiation and duration of breastfeeding: a randomised trial. *Br J Gen Pract, 56*(524), 191-197.

Nelson, K. A., Highstein, G. R., Garbutt, J., Trinkaus, K., Fisher, E. B., Smith, S. R., et al. (2011). A randomized controlled trial of parental asthma coaching to improve outcomes among urban minority children. *Archives of pediatrics & adolescent medicine, 165*(6), 520-526.

Nicholas, D. B., & Keilty, K. (2007). An evaluation of dyadic peer support for caregiving parents of children with chronic lung disease requiring technology assistance. *Soc Work Health Care, 44*(3), 245-259.

Olson, B. H., Haider, S. J., Vangjel, L., Bolton, T. A., & Gold, J. G. (2010). A quasi-experimental evaluation of a breastfeeding support program for low income women in Michigan. *Maternal and child health journal, 14*(1), 86-93.

Omer, K., Mhatre, S., Ansari, N., Laucirica, J., & Andersson, N. (2008). Evidence-based training of frontline health workers for door-to-door health promotion: a pilot randomized controlled cluster trial with Lady Health Workers in Sindh Province, Pakistan. *Patient education and counseling, 72*(2), 178-185.

Parent, N., & Fortin, F. (2000). A randomized, controlled trial of vicarious experience through peer support for male first-time cardiac surgery patients: impact on anxiety, self-efficacy expectation, and self-reported activity. *Heart & lung : the journal of critical care, 29*(6), 389-400.

Patterson, S. A., Ross-Edwards, B. M., & Gill, H. L. (2010). Stroke maintenance exercise group: pilot study on daily functioning in long-term stroke survivors. *Aust J Prim Health, 16*(1), 93-97.

Pearson, C. R., Micek, M. A., Simoni, J. M., Hoff, P. D., Matediana, E., Martin, D. P., et al. (2007). Randomized control trial of peer-delivered, modified directly observed therapy for HAART in Mozambique. *J Acquir Immune Defic Syndr, 46*(2), 238-244.

Preyde, M., & Ardal, F. (2003). Effectiveness of a parent "buddy" program for mothers of very preterm infants in a neonatal intensive care unit. *CMAJ : Canadian Medical Association journal = journal de l'Association medicale canadienne, 168*(8), 969-973.

Primomo, J., Johnston, S., DiBiase, F., Nodolf, J., & Noren, L. (2006). Evaluation of a community-based outreach worker program for children with asthma. *Public health nursing, 23*(3), 234-241.

Rahman, A., Malik, A., Sikander, S., Roberts, C., & Creed, F. (2008). Cognitive behaviour therapy-based intervention by community health workers for mothers with depression and their infants in rural Pakistan: a cluster-randomised controlled trial. *Lancet, 372*(9642), 902-909.

Rhodes, S. D., Hergenrather, K. C., Bloom, F. R., Leichliter, J. S., & Montano, J. (2009). Outcomes from a community-based, participatory lay health adviser HIV/STD prevention intervention for recently arrived immigrant Latino men in rural North Carolina. *AIDS education and prevention : official publication of the International Society for AIDS Education, 21*(5 Suppl), 103-108.

Riegel, B., & Carlson, B. (2004). Is individual peer support a promising intervention for persons with heart failure? *The Journal of cardiovascular nursing, 19*(3), 174-183.

Rowe, M., Bellamy, C., Baranoski, M., Wieland, M., O'Connell, M. J., Benedict, P., et al. (2007). A peer-support, group intervention to reduce substance use and criminality among persons with severe mental illness. *Psychiatr Serv, 58*(7), 955-961.

Sacco, W. P., Malone, J. I., Morrison, A. D., Friedman, A., & Wells, K. (2009). Effect of a brief, regular telephone intervention by paraprofessionals for type 2 diabetes. *Journal of behavioral medicine, 32*(4), 349-359.

Salzer, M. S., Palmer, S. C., Kaplan, K., Brusilovskiy, E., Ten Have, T., Hampshire, M., et al. (2010). A randomized, controlled study of Internet peer-to-peer interactions among women newly diagnosed with breast cancer. *Psychooncology, 19*(4), 441-446.

Simmons, D., Rush, E., & Crook, N. (2008). Development and piloting of a community health worker-based intervention for the prevention of diabetes among New Zealand Maori in Te Wai o Rona: Diabetes Prevention Strategy. *Public health nutrition, 11*(12), 1318-1325.

Simoni, J. M., Pantalone, D. W., Plummer, M. D., & Huang, B. (2007). A randomized controlled trial of a peer support intervention targeting antiretroviral medication adherence and depressive symptomatology in HIV-positive men and women. *Health Psychol, 26*(4), 488-495.

Smith, S. M., Paul, G., Kelly, A., Whitford, D. L., O'Shea, E., & O'Dowd, T. (2011). Peer support for patients with type 2 diabetes: cluster randomised controlled trial. *BMJ, 342*, d715.

Staten, L. K., Gregory-Mercado, K. Y., Ranger-Moore, J., Will, J. C., Giuliano, A. R., Ford, E. S., et al. (2004). Provider counseling, health education, and community health workers: the Arizona WISEWOMAN project. *Journal of women's health, 13*(5), 547-556.

Sullivan-Bolyai, S., Grey, M., Deatrick, J., Gruppuso, P., Giraitis, P., & Tamborlane, W. (2004). Helping other mothers effectively work at raising young children with type 1 diabetes. *The Diabetes educator, 30*(3), 476-484.

Travis, J., Roeder, K., Walters, H., Piette, J., Heisler, M., Ganoczy, D., et al. (2010). Telephone-based mutual peer support for depression: a pilot study. *Chronic Illn, 6*(3), 183-191.

Vilhauer, R. P., McClintock, M. K., & Matthews, A. K. (2010). Online support groups for women with metastatic breast cancer: a feasibility pilot study. *J Psychosoc Oncol, 28*(5), 560-586.

Weber, B. A., Roberts, B. L., Yarandi, H., Mills, T. L., Chumbler, N. R., & Wajsman, Z. (2007). The impact of dyadic social support on self-efficacy and depression after radical prostatectomy. *J Aging Health, 19*(4), 630-645.
