# Supplementary material for: Peer support of complex health behaviors in prevention and disease management with special reference to diabetes: systematic reviews
Source: Clin Diabetes Endocrinol. 2017 May 25;3:4. doi: 10.1186/s40842-017-0042-3 (PMC5471959; doi:10.1186/s40842-017-0042-3)
Supplement: Supplementary file 2 — Details of Studies Included in Review of Peer Support in Diabetes Management. Details of studies reviewed including population to which applied, whether intervention was for diabetes prevention or management, research design, characterization of strongest outcome measure and outcome observed, and pre and post Hemoglobin A1c values or changes in Hemoglobin A1c values. (DOCX 130 kb) [file 40842_2017_42_MOESM2_ESM.docx]

**Additional File 2 – Details of Studies Included in Review of Peer Support in Diabetes Management**

**Abreviations**

CRCT – Randomized Controlled Trial or Cluster Randomized Trial

M – Intervention for Diabetes Management

Nonsig – Nonsignificant Difference

Obj – Objective Measure of Strongest Outcome

OCD – Other Controlled Design

Other – other research design

P – Intervention for Diabetes Prevention

SBG – Significant Between-Group Difference Favoring Peer Support

Stand – Standardized Measure of Strongest Outcome

SWG – Significant Within-Group Change Favoring Peer Support

WGPP – Within-Group, Pre-Post Comparison

| Citation (references at bottom of document) | Country | Design | Prev / Mgmt | Strongest  Outcome | Measure of Strongest Outcome | Months from Pre to Post | Number of Peer Supporters | A1c Pre % | A1c Post % | Change in A1c % (if Pre and Post not reported) |
| --- | --- | --- | --- | --- | --- | --- | --- | --- | --- | --- |
| Assah, 2015 | Cameroon | Other | M | SBG | Obj | 6 | 96 | 9.6 | 6.6 |  |
| Auslander, 2002 | US | CRCT | P | SBG | Stand |  |  |  |  |  |
| Ayala, 2015 | US | CRCT | M | SBG | Obj | 12 | 122 | 8.7 | 8.3 |  |
| Babamoto, 2009 | US | CRCT | M | SBG | Stand | 6 | 75 | 8.6 | 7.2 |  |
| Chan, 2014 | China | CRCT | M | Nonsig | Obj | 12 | 296 |  |  | .3 |
| Collinsworth, 2013 | US | WGPP | M | SWG | Obj | 12 | 497 | 8.7 | 7.4 |  |
| Dale, 2009 | England | CRCT | M | SWG | Obj |  |  |  |  |  |
| Gagliardino, 2013 | Argentina | WGPP | M | SWG | Obj | 12 | 93 | 7.1 | 6.8 |  |
| Greenhalgh et al 2011 | England | CRCT | M | SBG | Stand | 12 | 51 | 8.2 | 7.6 |  |
| Heisler et al 2010 | US | CRCT | M | SBG | Obj | 6 | 113 | 8.02 | 7.73 |  |
| Islam, 2013 | US | WGPP | M | SWG | Stand | 12 | 14 | 7.6 | 7.1 |  |
| Micikas, 2015 | Guatemala | WGPP | M | SWG | Obj | 4 | 52 | 10.1 | 8.9 |  |
| Palmas, 2014 | US | CRCT | M | Nonsig | Obj | 12 | 178 | 8.77 | 8.42 |  |
| Prezio, 2013, 2014 | US | CRCT | M | SBG | Obj | 12 | 78 | 8.8 | 7.2 |  |
| Rothschild, 2014 | US | CRCT | M | SBG | Obj | 24 | 73 | 8.35 | 7.64 |  |
| Ruggiero, 2014 Af Am | US | CRCT | M | SWG | Stand | 12 | 69 | 9.09 | 9.01 |  |
| Ruggiero, 2014 Latino | US | CRCT | M | SWG | Stand | 12 | 65 | 7.76 | 7.86 |  |
| Ryabov, 2014 | US | OCD | M | SBG | Obj | 24 | 15 | 7.6 | 6.7 |  |
| Sacco, 2009 | US | CRCT | M | SBG | Stand | 6 | 27 | 8.4 | 7.4 |  |
| Safford, 2015 | US | CRCT | M | SBG | Obj | 15 |  |  |  |  |
| Shaya, 2014 | US | OCD | M | SBG | Obj | 6 | 68 |  |  | 0.81 |
| Simmons, 2008 | New Zealand | WGPP | P | SWG | Obj |  |  |  |  |  |
| Simmons, 2015 | England | CRCT | M | Nonsig | Obj | 12 | 781 | 7.5 | 7.5 |  |
| Smith, et al. 2011 | Ireland | CRCT | M | Nonsig | Obj | 24 | 187 | 7.2 | 7.1 |  |
| Sullivan-Bolyai, 2004 | US | CRCT | M | SBG | Stand |  |  |  |  |  |
| Tang, 2015 | US | CRCT | M | SBG | Obj | 15 | 7.8 |  |  | 0.5 |
| Thom, 2013 | US | CRCT | M | SBG | Obj | 6 | 148 |  |  | 1.07 |
| van der Wulp 2012 | Netherlands | CRCT | M | SWG | Stand | 6 |  |  |  |  |
| Willard-Grace 2015 | US | CRCT | M | SBG | Obj | 12 | 74 | 9.8 | 8.6 |  |
| Zhong, 2015 | China | OCD | M | SBG | Obj |  |  |  |  |  |

**References**

Assah, F. K., Atanga, E. N., Enoru, S., Sobngwi, E., & Mbanya, J. C. (2015). Community-based peer support significantly improves metabolic control in people with Type 2 diabetes in Yaounde, Cameroon. *Diabet Med, 32*(7), 886-889.

Auslander, W., Haire-Joshu, D., Houston, C., Rhee, C. W., & Williams, J. H. (2002). A controlled evaluation of staging dietary patterns to reduce the risk of diabetes in African-American women. *Diabetes Care, 25*(5), 809-814.

Ayala, G. X., Ibarra, L., Cherrington, A. L., Parada, H., Horton, L., Ji, M., et al. (2015). Puentes hacia una mejor vida (Bridges to a Better Life): Outcome of a Diabetes Control Peer Support Intervention. *Ann Fam Med, 13 Suppl 1*, S9-17.

Babamoto, K. S., Sey, K. A., Camilleri, A. J., Karlan, V. J., Catalasan, J., & Morisky, D. E. (2009). Improving diabetes care and health measures among hispanics using community health workers: results from a randomized controlled trial. *Health education & behavior : the official publication of the Society for Public Health Education, 36*(1), 113-126.

Chan, J. C., Sui, Y., Oldenburg, B., Zhang, Y., Chung, H. H., Goggins, W., et al. (2014). Effects of Telephone-Based Peer Support in Patients With Type 2 Diabetes Mellitus Receiving Integrated Care: A Randomized Clinical Trial. *JAMA Intern Med, 174*(6), 972-981.

Collinsworth, A. W., Vulimiri, M., Schmidt, K. L., & Snead, C. A. (2013). Effectiveness of a community health worker-led diabetes self-management education program and implications for CHW involvement in care coordination strategies. *Diabetes Educ, 39*(6), 792-799.

Dale, J., Caramlau, I., Sturt, J., Friede, T., & Walker, R. (2009). Telephone peer-delivered intervention for diabetes motivation and support: the telecare exploratory RCT. *Patient Education and Counseling, 75*(1), 91-98.

Gagliardino, J. J., Lapertosa, S., Pfirter, G., Villagra, M., Caporale, J. E., Gonzalez, C. D., et al. (2013). Clinical, metabolic and psychological outcomes and treatment costs of a prospective randomized trial based on different educational strategies to improve diabetes care (PRODIACOR). *Diabet Med, 30*(9), 1102-1111.

Greenhalgh, T., Campbell-Richards, D., Vijayaraghavan, S., Collard, A., Malik, F., Griffin, M., et al. (2011). New models of self-management education for minority ethnic groups: pilot randomized trial of a story-sharing intervention. *Journal of health services research & policy, 16*(1), 28-36.

Heisler, M., Vijan, S., Makki, F., & Piette, J. D. (2010). Diabetes control with reciprocal peer support versus nurse care management: a randomized trial. *Annals of Internal Medicine, 153*(8), 507-515.

Islam, N. S., Wyatt, L. C., Patel, S. D., Shapiro, E., Tandon, S. D., Mukherji, B. R., et al. (2013). Evaluation of a community health worker pilot intervention to improve diabetes management in Bangladeshi immigrants with type 2 diabetes in New York City. *Diabetes Educ, 39*(4), 478-493.

Micikas, M., Foster, J., Weis, A., Lopez-Salm, A., Lungelow, D., Mendez, P., et al. (2015). A Community Health Worker Intervention for Diabetes Self-Management Among the Tz'utujil Maya of Guatemala. *Health Promot Pract, 16*(4), 601-608.

Palmas, W., Findley, S. E., Mejia, M., Batista, M., Teresi, J., Kong, J., et al. (2014). Results of the northern Manhattan diabetes community outreach project: a randomized trial studying a community health worker intervention to improve diabetes care in Hispanic adults. *Diabetes Care, 37*(4), 963-969.

Prezio, E. A., Balasubramanian, B. A., Shuval, K., Cheng, D., Kendzor, D. E., & Culica, D. (2014). Evaluation of quality improvement performance in the Community Diabetes Education (CoDE) program for uninsured Mexican Americans: results of a randomized controlled trial. *Am J Med Qual, 29*(2), 124-134.

Prezio, E. A., Cheng, D., Balasubramanian, B. A., Shuval, K., Kendzor, D. E., & Culica, D. (2013). Community Diabetes Education (CoDE) for uninsured Mexican Americans: a randomized controlled trial of a culturally tailored diabetes education and management program led by a community health worker. *Diabetes Res Clin Pract, 100*(1), 19-28.

Rothschild, S. K., Martin, M. A., Swider, S. M., Tumialan Lynas, C. M., Janssen, I., Avery, E. F., et al. (2014). Mexican American trial of community health workers: a randomized controlled trial of a community health worker intervention for Mexican Americans with type 2 diabetes mellitus. *Am J Public Health, 104*(8), 1540-1548.

Ruggiero, L., Riley, B. B., Hernandez, R., Quinn, L. T., Gerber, B. S., Castillo, A., et al. (2014). Medical assistant coaching to support diabetes self-care among low-income racial/ethnic minority populations: randomized controlled trial. *West J Nurs Res, 36*(9), 1052-1073.

Ryabov, I. (2014). Cost-effectiveness of Community Health Workers in controlling diabetes epidemic on the U.S.-Mexico border. *Public Health, 128*(7), 636-642.

Sacco, W. P., Malone, J. I., Morrison, A. D., Friedman, A., & Wells, K. (2009). Effect of a brief, regular telephone intervention by paraprofessionals for type 2 diabetes. *J Behav Med, 32*(4), 349-359.

Safford, M. M., Andreae, S., Cherrington, A. L., Martin, M. Y., Halanych, J., Lewis, M., et al. (2015). Peer Coaches to Improve Diabetes Outcomes in Rural Alabama: A Cluster Randomized Trial. *Ann Fam Med, 13 Suppl 1*, S18-26.

Shaya, F. T., Chirikov, V. V., Howard, D., Foster, C., Costas, J., Snitker, S., et al. (2014). Effect of social networks intervention in type 2 diabetes: a partial randomised study. *J Epidemiol Community Health, 68*(4), 326-332.

Simmons, D., Prevost, A. T., Bunn, C., Holman, D., Parker, R. A., Cohn, S., et al. (2015). Impact of Community Based Peer Support in Type 2 Diabetes: A Cluster Randomised Controlled Trial of Individual and/or Group Approaches. *PLoS One, 10*(3), e0120277.

Simmons, D., Rush, E., & Crook, N. (2008). Development and piloting of a community health worker-based intervention for the prevention of diabetes among New Zealand Maori in Te Wai o Rona: Diabetes Prevention Strategy. *Public health nutrition, 11*(12), 1318-1325.

Smith, S. M., Paul, G., Kelly, A., Whitford, D. L., O'Shea, E., & O'Dowd, T. (2011). Peer support for patients with type 2 diabetes: cluster randomised controlled trial. *BMJ, 342*, d715.

Sullivan-Bolyai, S., Grey, M., Deatrick, J., Gruppuso, P., Giraitis, P., & Tamborlane, W. (2004). Helping other mothers effectively work at raising young children with type 1 diabetes. *Diabetes Educ, 30*(3), 476-484.

Tang, T. S., Funnell, M. M., Sinco, B., Spencer, M. S., & Heisler, M. (2015). Peer-Led, Empowerment-Based Approach to Self-Management Efforts in Diabetes (PLEASED): A Randomized Controlled Trial in an African American Community. *Ann Fam Med, 13 Suppl 1*, S27-35.

Thom, D. H., Ghorob, A., Hessler, D., De Vore, D., Chen, E., & Bodenheimer, T. A. (2013). Impact of peer health coaching on glycemic control in low-income patients with diabetes: a randomized controlled trial. *Annals of family medicine, 11*(2), 137-144.

van der Wulp, I., de Leeuw, J. R., Gorter, K. J., & Rutten, G. E. (2012). Effectiveness of peer-led self-management coaching for patients recently diagnosed with Type 2 diabetes mellitus in primary care: a randomized controlled trial. *Diabetic medicine : a journal of the British Diabetic Association, 29*(10), e390-397.

Willard-Grace, R., Chen, E. H., Hessler, D., DeVore, D., Prado, C., Bodenheimer, T., et al. (2015). Health coaching by medical assistants to improve control of diabetes, hypertension, and hyperlipidemia in low-income patients: a randomized controlled trial. *Ann Fam Med, 13*(2), 130-138.

Zhong, X., Wang, Z., Fisher, E. B., & Tanasugarn, C. (2015). Peer Support for Diabetes Management in Primary Care and Community Settings in Anhui Province, China. *Ann Fam Med, 13 Suppl 1*, S50-58.
